# Functional Materials for the Remediation of Arsenic-Contaminated Soils: A Review

**DOI:** 10.3390/toxics14080733

**Published:** 2026-08-20

**Authors:** Chenqi Peng, Bowen Wang, Han Cheng, Ning Li, Yuhong Su

**Affiliations:** 1 College of Chemical Engineering, Xinjiang University, Urumqi 830046, China; 2 Key Laboratory of Oil and Gas Fine Chemicals, Ministry of Education & Xinjiang Uygur Autonomous Region, Xinjiang University, Urumqi 830046, China; 3 Institute of Agricultural Resources and Environment, Xinjiang Academy of Agricultural Sciences, Urumqi 830091, China; 4 Key Laboratory of Northwest Oasis Agriculture Environment, Ministry of Agriculture, Urumqi 830091, China

**Keywords:** arsenic contamination, soil remediation, functional materials, remediation performance

## Abstract

Soil arsenic contamination is an increasingly serious global environmental problem that poses substantial risks to environmental safety and human health. With ongoing advances in green remediation technologies, functional materials have become important for the remediation of arsenic-contaminated soils because of their tailored remediation properties and potential for engineering application. This review systematically examines the remediation performance of functional materials derived from carbon-based materials, natural polymers, metals and metal oxides, and industrial solid wastes. The performance characteristics, modification approaches, and arsenic immobilization mechanisms are also summarized. Finally, recommendations and future directions for the development of functional materials for arsenic-contaminated soil remediation are provided to guide further research.

## 1. Introduction

Arsenic (As) is a naturally occurring metalloid that is highly toxic to a wide range of organisms, including humans, and is recognized as a contaminant of global concern [1,2]. Human exposure to arsenic can occur through inhalation and the consumption of contaminated drinking water and food, particularly rice and meat [3,4]. Such exposure may cause chronic cardiovascular, neurological, renal, and respiratory diseases. Long-term arsenic exposure is also associated with an increased risk of bladder, skin, and liver cancers. Accordingly, the International Agency for Research on Cancer (IARC) has classified inorganic arsenic compounds as Group 1 carcinogens [5,6,7].

Arsenic contamination originates from both anthropogenic activities and natural processes. Industrial activities, including the mining and processing of arsenic-bearing minerals and the combustion of fossil fuels, can release arsenic into the environment. In addition, the production and use of arsenic-containing products, such as wood preservatives, pesticides, and alloys, further contribute to arsenic contamination. Natural arsenic inputs are mainly associated with volcanic eruptions, rock weathering, and geothermal activity [8,9]. Soil arsenic contamination is an increasingly serious global issue, particularly in China, South Asia, Southeast Asia, and central South America [10]. India, Bangladesh, Thailand, and Cambodia have long experienced soil arsenic contamination, primarily because of local geological conditions and the use of arsenic-contaminated irrigation water [10,11]. It has been projected that the mean arsenic concentration in Chinese soils may reach 13.6 mg kg^−1^ by 2040. Contamination is expected to be more severe in southern China, where 18.3% of rice may be cultivated in soils with arsenic concentrations exceeding 20 mg kg^−1^ [12].

To address the increasingly severe problem of arsenic contamination, various remediation approaches have been developed, including chemical, physical, biological, and integrated remediation technologies [13]. However, these approaches have several limitations, such as soil organic matter loss, disruption of soil biota, potential secondary contamination, high costs, and long remediation periods. Driven by advances in green and sustainable remediation technologies, functional materials have received increasing attention in the remediation of arsenic-contaminated soils because of their targeted remediation capability and practical applicability. In recent years, several reviews have examined arsenic-contaminated soil remediation from different perspectives, including the life cycle of arsenic contamination and sustainable remediation technologies [1], advances in arsenic removal technologies [14], management strategies for nanomaterial-based remediation of arsenic contamination [15], progress in organic and inorganic stabilization materials for arsenic remediation [16], and mechanisms of heavy metal immobilization by iron-based materials [17]. However, most existing reviews focus on a single material class or a specific remediation pathway. Systematic comparisons of different functional materials for arsenic-contaminated soil remediation remain limited, and key issues, including material modification strategies, synergistic effects among multiple mechanisms, cost-effectiveness, and engineering feasibility, have not been sufficiently addressed [18]. Based on a comprehensive search and review of the published literature, this review summarizes recent advances in six major categories of functional materials for arsenic-contaminated soil remediation: carbon-based materials, natural polymer materials, metals and their oxides, industrial solid waste-based materials, clay minerals, and molecular sieves. These materials are systematically evaluated in terms of preparation methods, remediation mechanisms, removal efficiency, advantages, and limitations. Accordingly, future research directions are proposed to address unresolved scientific questions and to support the development and engineering application of materials for arsenic-contaminated soil remediation.

## 2. Carbon-Based Functional Materials

Carbon-based functional materials are multifunctional materials composed mainly of carbon. Their low cost, environmental compatibility, and renewability make them sustainable options for the remediation of arsenic-contaminated soils [19,20].

### 2.1. Biochar-Based Functional Materials

**Figure 1:**
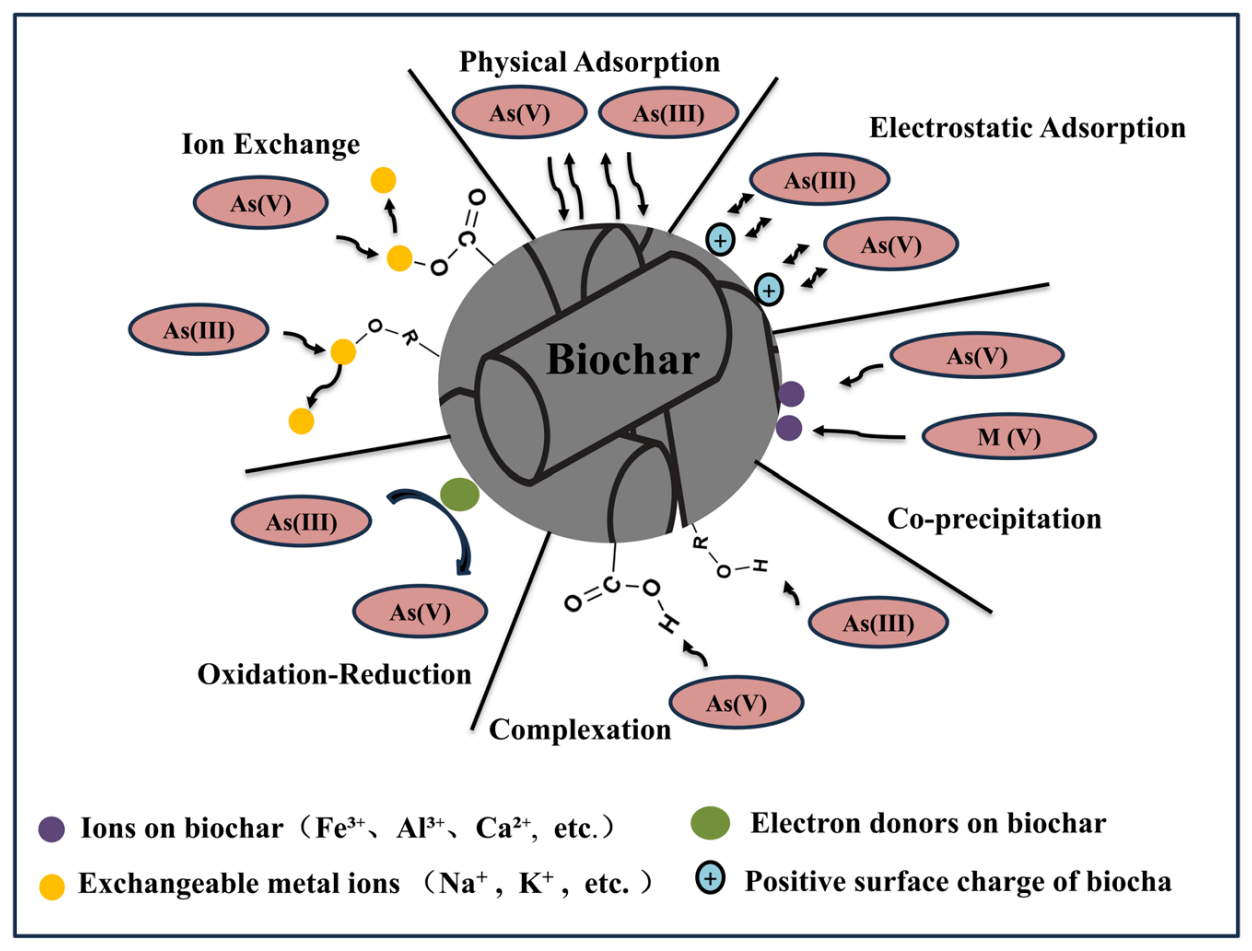
Schematic illustration of arsenic adsorption mechanisms on biochar.

Carbon-based materials are among the most extensively studied functional materials for the remediation of arsenic-contaminated soils. Among these materials, biochar has received considerable attention because of its low cost and tunable physicochemical properties. Biochar is produced through the oxygen-limited pyrolysis of agricultural, forestry, and other organic wastes. It generally has a high specific surface area and abundant surface functional groups. Biochar immobilizes heavy metals in soil through adsorption, complexation, and ion exchange (Figure 1), thereby reducing their mobility and bioavailability. In addition, biochar can improve soil structure and enhance water- and nutrient-holding capacity, contributing to arsenic immobilization and improved agricultural productivity [21,22].

The physicochemical properties of biochar, including specific surface area, pore structure, surface functional groups, and ash composition, are strongly influenced by feedstock type and pyrolysis conditions. In general, increasing pyrolysis temperature increases the specific surface area and aromaticity of biochar but decreases the abundance of oxygen-containing functional groups. Biochars derived from woody feedstocks usually have higher carbon contents and specific surface areas than those produced from herbaceous biomass or manure. In contrast, biochars derived from herbaceous biomass and manure are generally richer in mineral ash, which can supply additional alkaline sites and nutrient elements to the soil [23]. These properties directly influence the arsenic adsorption capacity and immobilization mechanisms of biochars produced from different feedstocks. Therefore, the selection of biochar-based remediation materials should take into account the feedstock source, pyrolysis conditions, and properties of the target contaminated site. To improve the efficiency and field applicability of biochar for arsenic-contaminated soil remediation, various modification strategies have been developed, including structural optimization, surface functionalization, metal loading, and microbial augmentation (Table 1).

#### 2.1.1. Structural Optimization of Biochar

Structural optimization aims to develop a porous architecture and increase the porosity and surface roughness of biochar. Common approaches include activation and mechanical modification. Chemical and gas activation are widely used activation methods. Oxidants, acids, and alkalis can modify the pore structure and surface properties of biochar. They can also alter its surface charge, thereby improving adsorption performance [24]. KOH treatment significantly increased the specific surface area of biochar and enhanced its arsenic adsorption capacity [25]. KMnO_4_ oxidation altered the pH and organic matter content of biochar. The resulting modified biochar reduced arsenic release from soil by 77%, thus decreasing the associated environmental risk [26]. Gas activation uses steam, ozone, or other activating gases at high temperatures to selectively remove amorphous carbon components from biochar. This process opens micropores and increases the specific surface area [27]. One study reported that gas activation significantly increased the specific surface area and porosity of biochar, resulting in a maximum As(V) adsorption capacity 7.7 times higher than that of pristine biochar [28].

Ball milling is a promising mechanical modification method that reduces the particle size of biochar [29]. Ball-milled biochar can rapidly remove As(V) from water because of its increased specific surface area [30]. Ultrasonic treatment can remove impurities from internal pores, thereby increasing the porosity and specific surface area of biochar [31]. Luo et al. [32] treated biochar with 28 kHz ultrasound and reported an 18.6-fold increase in specific surface area, together with a marked improvement in As(V) adsorption. Ultraviolet irradiation and freeze–thaw cycling have also been used to modify biochar-based materials. Overall, chemical activation is relatively simple to perform and can substantially improve the properties of biochar, including its porosity and specific surface area. However, it requires chemical reagents, such as acids and alkalis, as well as subsequent washing, which increases production costs and generates additional environmental impacts. Gas activation requires more specialized equipment but produces less chemical waste during processing. Ball milling does not require chemical reagents and is suitable for large-scale production; however, it is less effective than chemical activation in developing the pore structure of biochar. Ultrasonic treatment can also enhance porosity and specific surface area by removing impurities from internal pores. Therefore, the modification method should be selected according to site-specific conditions and the functional requirements of the material.

#### 2.1.2. Surface Functional Group Modification of Biochar

The surface properties of biochar-based materials depend largely on the type and abundance of surface functional groups [33]. Modification of cotton-stalk biochar with H_3_PO_4_ and KOH increased the abundance of hydroxyl and amino groups and enhanced arsenic adsorption [34]. Organic compounds can also increase the abundance of existing surface functional groups or introduce new functional groups. Wang et al. [35] modified magnetic biochar derived from traditional Chinese medicine residues with chitosan to prepare CMBC. X-ray photoelectron spectroscopy (XPS) showed that the contents of amino, hydroxyl, carboxyl, and other functional groups on the CMBC surface increased significantly. L-Cysteine modification introduced thiol groups onto biochar and increased its As(V) adsorption capacity by approximately 29% [36]. The incorporation of non-metal heteroatoms, including sulfur, nitrogen, and silicon, can regulate the surface properties of biochar and increase the abundance of functional groups. An Fe/S co-doping strategy was used to prepare an Fe/S-BC composite. Sulfur was present on the biochar surface in multiple oxidation states, which substantially improved As (III) adsorption [23,37]. With respect to arsenic removal, functional-group modification primarily enhances surface complexation and ligand exchange. Oxygen-containing groups, including hydroxyl (-OH) and carboxyl (-COOH) groups, can form inner-sphere or outer-sphere complexes with arsenate and arsenite species. The introduction of thiol (-SH) groups enhances the adsorption of As(V), while amino (-NH_2_) groups provide additional coordination sites that improve the adsorption of As(III). Notably, functional-group modification generally has a smaller effect on the specific surface area of biochar than structural modification. Its principal advantage lies in enhancing the specific affinity of biochar for arsenic species.

#### 2.1.3. Construction of Metal Active Sites on Biochar

Pristine biochar generally has limited arsenic adsorption capacity. Introducing active sites based on metals such as iron and manganese can improve arsenic adsorption and immobilization [38]. Song and co-workers prepared biochar-supported nanoscale zero-valent iron (nZVI@BC) for the remediation of arsenic-contaminated soil. Simulation experiments showed that nZVI@BC significantly reduced the readily soluble arsenic fraction in soil [39]. In another study, magnetic iron oxide-modified mulberry branch biochar (MBC) was prepared. The addition of 5% MBC increased the residual arsenic fraction by 161%, thereby effectively reducing arsenic mobility [40]. The mechanisms responsible for arsenic immobilization by iron-based modifications mainly involve ligand exchange, coprecipitation, and electrostatic attraction. Hydroxyl groups on the surfaces of Fe(III) oxides and hydroxides undergo ligand exchange with arsenate to form inner-sphere surface complexes. In addition, Fe^2+^ released during the corrosion of zero-valent iron can be oxidized to Fe^3+^, which subsequently participates in coprecipitation with arsenic to form sparingly soluble minerals such as FeAsO_4_. The positive surface charge of iron oxides can also enhance the adsorption of negatively charged arsenate through electrostatic attraction.

Unlike iron-based modification, which immobilizes arsenic mainly through surface complexation, coprecipitation, and electrostatic attraction, manganese-based modification introduces redox-active sites into biochar [41]. MnO_2_-loaded biochar can promote arsenic immobilization through several mechanisms. First, MnO_2_ loading can improve the pore structure and expose reactive sites. Second, oxygen-containing surface functional groups, including -COOH and -OH, can act as electron donors and enhance the oxidation of As(III) by MnO_2_. Third, Mn(II) generated during the reaction can combine with As (V) to form sparingly soluble manganese arsenates, such as MnHAsO_4_·H_2_O. The porous biochar framework can provide space for the retention of these precipitates [42,43]. Manganese oxide-modified biochar significantly inhibited arsenic uptake by rice, reducing arsenic concentrations in roots, leaves, and grains by 65.4%, 44.8%, and 19.8%, respectively [44].

Fe-Mn materials have high reactivity and large specific surface areas and are widely used for arsenic immobilization. However, these materials are prone to aggregation. Loading Fe-Mn materials onto biochar is therefore an effective approach to improve their dispersion [45]. A soil amendment experiment showed that Fe-Mn modification changed the physicochemical properties of biochar and increased the contents of soil organic matter, ammonium nitrogen, and available phosphorus. Functional groups on the modified biochar provided sites for arsenic transformation and adsorption. They also increased the electrostatic attraction of soil colloids to arsenic, leading to a higher proportion of arsenic in the residual fraction [46]. Other metals have also been investigated for the construction of reactive sites on biochar. In a pot experiment, zirconium-modified biochar (BC-Zr) reduced arsenic concentrations in cowpea stems and leaves by 30.8% and 42.6%, respectively, relative to the control. Its remediation performance was better than that of iron- and titanium-modified biochars [47]. Another pot experiment showed that Fe-Mn-Ce ternary-modified biochar reduced the bioavailable fraction of soil arsenic by 10.23% [48]. Constructing metal-based active sites is one of the most effective approaches for improving the capacity of biochar to immobilize arsenic. Iron-based modification has been studied most extensively, whereas manganese-based modification has distinct advantages in the oxidation of As(III). Fe-Mn bimetallic and multimetallic modifications can further improve arsenic remediation through synergistic effects. However, these modifications involve more complex preparation procedures and higher costs; therefore, their remediation performance and economic feasibility should be balanced in practical applications.

#### 2.1.4. Biochar–Microbe Composite Materials

Biochar–microbe composite materials combine the advantages of biochar and microorganisms to improve arsenic removal and immobilization. Biochar provides a suitable habitat for microorganisms and can act as an electron shuttle in biogeochemical reactions. Microorganisms can promote arsenic speciation transformation through co-metabolic processes, thereby reducing arsenic mobility and toxicity. These interactions can improve microbial tolerance to environmental stress and enhance arsenic remediation efficiency [49,50]. Ji et al. [51] immobilized microorganisms on biochar through physical adsorption and sodium alginate encapsulation. The resulting composite achieved an arsenic immobilization efficiency of 74.77%. It also enhanced soil enzyme activity and microbial metabolism, thereby promoting arsenic stabilization. In another study, Bacillus sp. K1 was loaded onto magnetic biochar. The composite promoted iron oxide formation and achieved efficient arsenic adsorption, with an As(III) adsorption capacity of 4.58 mg g^−1^ [52]. The major scientific challenges are as follows: (1) the survival and colonization of introduced microorganisms in complex soil environments remain poorly understood; (2) the long-term interactions among microorganisms, biochar, and arsenic have yet to be fully elucidated; and (3) inoculation outcomes at the field scale differ substantially from those observed under laboratory conditions, while long-term field validation data remain limited.

**Table 1:** Comparison of different modification strategies for biochar.

Strategy	Structural Optimization	Functional Group Modification	Construction of Metal Active Sites	Microbial Synergy
Representative methods	KOH activation, gas activation, ball milling, ultrasonication	Organic grafting, heteroatom doping	Fe/Mn/Fe-Mn/Zr loading	Physical adsorption/sodium alginate encapsulation for microorganism immobilization
Modification mechanism	Increase specific surface area and porosity, improve pore structure	Introduce O/N/S-containing functional groups, enhance surface complexation and ligand exchange	Ligand exchange, coprecipitation, electrostatic attraction, oxidation; synergistic effects in multi-metal systems	Biotransformation, biochar adsorption
As removal efficiency enhancement	Moderate–high (gas activation: 7.7-fold increase in As(V) adsorption capacity; ultrasonication: 18.6-fold increase in specific surface area)	Moderate (thiol modification increases As(V) adsorption by ~29%; Fe/S co-doping significantly enhances As(III) adsorption)	High (residual As fraction increased by 161%; bioavailable As decreased by ~10%; rice grain As decreased by up to 19.8%)	Moderate–high (immobilization efficiency 74.77%; As(III) adsorption capacity 4.58 mg/g)
Advantages	Relatively simple operation; enhanced physical adsorption; does not alter biochar chemical composition	Provides selective recognition capability; can be tailored for specific As species	Multiple mechanisms synergy; highest efficiency; magnetic materials are recoverable	Environmentally friendly; sustainable As speciation conversion; enhances soil enzyme activity and microbial metabolic function
Limitations	Poor selectivity for physical adsorption; susceptible to competing ions and pH; chemical activation requires acids/alkalis and washing steps, increasing environmental burden	High cost of some modifiers; poor thermal stability; weaker effect on specific surface area compared to structural optimization	Metal leaching risk; complex preparation for multi-metal systems; nanoparticle aggregation	Long remediation period; unclear survival and colonization of introduced microorganisms; limited long-term field validation data
Preparation complexity	Low–moderate	Moderate–high	Moderate–high	Moderate
Cost	Low–moderate	Moderate–high	Moderate	Moderate
Applicable scenarios	Low-concentration As contamination, scenarios with few competing ions	Selective removal of specific As species (e.g., As(V))	High-concentration As contamination; coexistence of multiple As species	Long-term remediation; ecologically sensitive areas
Ref.	[24,25,26,27,28,29,30,31,32]	[33,34,35,36,37]	[38,39,40,41,42,43,44,45,46,47,48]	[49,50,51]

### 2.2. Graphene-Based Functional Materials

Graphene is a two-dimensional carbon material with a hexagonal honeycomb lattice. Graphene-based materials, particularly graphene oxide (GO), can possess a large specific surface area and abundant oxygen-containing functional groups, including carboxyl, hydroxyl, and epoxy groups. These properties enable the removal of various environmental contaminants, including arsenic [53,54]. Akhtar et al. [55] prepared two graphene oxide-based materials, GO-C and bGO-P, by chemical exfoliation and pyrolysis, respectively. Both materials contained abundant surface functional groups and showed interactions with arsenic species. Soil remediation experiments showed that GO-C and bGO-P achieved arsenic removal efficiencies of 84.8–98.5%, indicating effective arsenic immobilization. In contrast, nano-graphene oxide (nGOx) increased arsenic bioavailability by 64%, which was attributed to electrostatic repulsion between negatively charged surface functional groups and arsenate species. This characteristic may be useful in phytoremediation strategies that require enhanced arsenic mobility [56].

Recent efforts to improve the arsenic adsorption capacity of graphene-based materials have focused mainly on metal oxide functionalization and three-dimensional structural design [57]. Gao et al. [58] prepared a magnetic graphene-loaded biochar gel (FeBG) and evaluated its performance in a soil incubation experiment using arsenic (As)- and antimony (Sb)-co-contaminated soil from a mining area. FeBG had a specific surface area of 80.5 m^2^/g, significantly higher than that of pristine biochar (57.4 m^2^/g), and exhibited newly formed Fe-O bonds. Application of FeBG decreased the concentration of Na_2_HPO_4_-extractable As in soil by 9.9%. Its immobilization performance was attributed to the combined effects of electrostatic attraction, surface complexation, and π–π interactions.

Nevertheless, the use of graphene-based nanomaterials for soil remediation remains challenging. Graphene and graphene oxide may exhibit high mobility in soil, and their nanosheets can migrate with water flow or soil pore water to deeper soil layers or groundwater, raising concerns regarding their environmental transport and fate. Soil application of graphene oxide can significantly alter microbial communities within one week and cause a transient decline in biodiversity [59,60]. In addition, the production costs of graphene-based materials remain substantially higher than those of biochar and most clay minerals, which limits their large-scale application. Therefore, graphene-based materials should be primarily considered for high-risk, small-area contaminated sites requiring rapid treatment, rather than for large-scale remediation of agricultural soils where low-cost materials are more suitable. Future material design should seek to reduce production costs and environmental impacts, and rigorous ecotoxicological assessments are needed to establish their environmental safety.

### 2.3. Hydrochar-Based Functional Materials

Hydrochar is a carbon-rich material produced from biomass through hydrothermal carbonization (HTC) at relatively mild temperatures. Unlike biochar produced by pyrolysis, HTC can directly process wet biomass without pre-drying. It generally operates at lower temperatures, requires less energy, and provides higher solid yields. Because no drying step is required, HTC may also have a lower environmental footprint, although the process water requires appropriate management. Hydrochar is rich in oxygen-containing functional groups and may contain soluble organic constituents, which contribute to its adsorption capacity and reactivity [18,61]. The immobilization efficiency of hydrochar is strongly influenced by feedstock type and carbonization temperature. Process parameters, including temperature, residence time, and the water-to-biomass ratio, also affect its physicochemical properties and potential for environmental applications [62]. In a pot experiment, a 2% hydrochar amendment achieved an As immobilization rate of 71.62% in alkaline soil [63]. Modification can further improve the As remediation performance of hydrochar. Iron- and manganese-modified hydrochar (FMHC) reduced the leached As concentration by more than 94% [64]. At an application rate of 1%, phosphate rock-modified hydrochar (RP-MHC) achieved an As stabilization rate of 63.44% in soil and significantly increased soil cation-exchange capacity, organic matter content, and nutrient concentrations [65].

Hydrochar and biochar differ substantially in their production pathways and physicochemical properties [66]. Biochar is produced by high-temperature pyrolysis and generally has a higher degree of carbonization, larger specific surface area, and greater aromaticity. These properties favor the physical adsorption of contaminants. In contrast, hydrochar is produced by lower-temperature HTC and typically has a lower degree of carbonization and a smaller specific surface area. However, it retains more oxygen-containing functional groups, such as hydroxyl (-OH), carboxyl (-COOH), and carbonyl (C=O) groups, resulting in greater hydrophilicity and surface complexation capacity. For As removal, biochar mainly relies on physical adsorption sites associated with its large specific surface area and, after metal modification, on ligand exchange and coprecipitation. Hydrochar relies more on surface complexation and electrostatic interactions between surface oxygen-containing functional groups and arsenate or arsenite species [67]. The main limitations of hydrochar include its relatively low specific surface area and limited long-term structural stability. In addition, HTC process water contains dissolved organic matter and nutrients and should be properly treated to prevent secondary pollution. Unprocessed biomass, hydrochar, and pyrolytic biochar should be regarded as complementary materials. Their selection should be based on contaminant characteristics, the required remediation period, and economic and environmental constraints [18].

## 3. Natural Polymer-Based Functional Materials

Natural polymers are widely used in environmental remediation because they are abundant, inexpensive, and rich in reactive functional groups. Natural polymer-based functional materials can remove arsenic through coordination, ion exchange, and electrostatic interactions. Their readily modifiable structures also provide considerable scope for functionalization [68].

### 3.1. Chitosan-Based Materials

Chitosan is obtained by the deacetylation of chitin. Its molecular structure contains abundant reactive functional groups, particularly hydroxyl and amino groups, which provide binding sites for pollutant adsorption and chemical modification. Arsenic adsorption by chitosan is mainly attributed to electrostatic attraction between protonated amino groups (-NH_3_^+^) and arsenate anions, as well as coordination interactions involving hydroxyl and amino groups. Under acidic conditions, extensive protonation of amino groups enhances the adsorption of As(V). However, under neutral to alkaline conditions, amino-group deprotonation decreases the positive charge of chitosan and markedly reduces its adsorption capacity. This pH-dependent adsorption behavior presents an inherent limitation. Although acidic conditions favor As adsorption, they also promote the protonation and dissolution of chitosan. In addition, the limited adsorption capacity and insufficient rigidity of chitosan chains restrict its practical performance. Therefore, modification is required to increase the density of coordination-active sites and improve resistance to swelling [69,70,71]. Incorporation of iron-based materials can substantially improve the environmental stability of chitosan-based composites. A composite prepared from industrial waste iron filings and chitosan achieved an As immobilization efficiency of 73.98% and effectively reduced the bioavailability of soil As [72]. Similarly, a biochar–chitosan composite (BCC) applied to soil contaminated with iron mine tailings reduced bioavailable As by 85% and maintained an immobilization efficiency above 75% after three regeneration cycles [73]. In As-contaminated paddy soil, chitosan-based silicon nanoparticles (CBSNs) reduced As uptake by rice roots by 35–43% and decreased As concentrations in rice grains by 36%. They also altered the structure of the rhizosphere microbial community [74].

Despite these advantages, several factors limit the practical application of chitosan-based materials. In acidic soils, chitosan is susceptible to protonation-induced dissolution. Crosslinking can improve its chemical stability and specific surface area; however, repeated regeneration often reduces the density of adsorption sites, resulting in limited reusability [75,76]. Chitosan is mainly derived from shrimp and crab shell waste, but its laboratory-scale synthesis cost is substantially higher than that of cellulose-based materials. Moreover, complex modification procedures reduce its economic feasibility for large-scale farmland remediation compared with low-cost amendments such as biochar [77]. The complex speciation of As in soil, stringent material-modification requirements, and limited cost-effectiveness restrict the wider application of chitosan-based materials. Future studies should focus on developing efficient and cost-effective chitosan-based systems for As immobilization in soil.

### 3.2. Cellulose-Based Materials

Cellulose is a naturally occurring polysaccharide, a major component of plant cell walls, and the most abundant biopolymer on Earth [78]. It has abundant surface hydroxyl groups, good mechanical strength, and high potential for structural modification, making it a promising material for environmental remediation [79,80]. However, pristine cellulose has limited capacity for direct adsorption of arsenic oxyanions. At neutral pH, its surface hydroxyl groups are not positively charged and therefore have limited ability to capture arsenate through electrostatic attraction. Consequently, cellulose is mainly used as a support matrix in arsenic remediation. Positively charged or arsenic-affinitive components, such as iron or manganese oxides and amino-functional groups, can be introduced onto the cellulose surface or into its framework to enhance arsenic removal [81]. For example, Shil et al. prepared an adsorbent with high selectivity for As(III) by modifying nanocellulose with iron and zirconium [82]. Carboxymethyl cellulose (CMC)-stabilized nanoscale schwertmannite achieved an immobilization efficiency exceeding 99.9% for inorganic As in soil [83]. Su et al. [84] prepared a membrane material by loading manganese-doped hydrous iron oxide onto cellulose and coating it with poly(vinyl alcohol). In contaminated-soil remediation experiments, application of the composite membrane at 0.3 wt% reduced the concentration of toxicity characteristic leaching procedure (TCLP)-extractable As by 65%, indicating effective As immobilization and good material stability. Cellulose is widely available, inexpensive, and supported by relatively mature processing technologies. It therefore has strong potential for large-scale production and is the most economically favorable among the three natural polymers [85].

### 3.3. Lignin-Based Materials

Lignin is a three-dimensional aromatic biopolymer formed by the oxidative coupling of phenylpropanoid monolignols, including *p*-coumaryl alcohol, coniferyl alcohol, and sinapyl alcohol. Its structure contains various ether and carbon–carbon linkages. Lignin is amorphous, highly crosslinked, rich in functional groups, and characterized by a rigid aromatic backbone, which confers chemical stability and amenability to modification [86,87]. It contains aromatic rings, phenolic hydroxyl groups, aliphatic hydroxyl groups, and ether linkages that can bind metal ions through complexation and related surface interactions. However, lignin has a limited affinity for anionic arsenic species. Under neutral to alkaline conditions, its abundant phenolic hydroxyl groups (-OH) undergo deprotonation, causing the lignin surface to become negatively charged and thereby inhibiting arsenic adsorption. In addition, lignin lacks metal-containing active sites that can form strong inner-sphere complexes with arsenate. Its native functional groups, including phenolic and aliphatic hydroxyl groups, interact with arsenic mainly through relatively weak hydrogen bonding and outer-sphere complexation. These interactions provide limited adsorption affinity. The amorphous structure of lignin also results in limited specific surface area and porosity, which hinder arsenate diffusion and adsorption [88,89]. Common modification strategies include doping, grafting, and ultrasonication [90]. These strategies mainly aim to alter the surface charge of lignin or introduce additional active sites. For example, an iron oxide–modified lignin soil amendment (LnFe50) markedly reduced arsenic leaching at an application rate of 5%. Mechanistic analysis showed that arsenic formed stable surface complexes with the iron oxide component through surface complexation [91]. Researchers also prepared a lignin-based adsorbent, Ca@N-Lig, through nitrogen doping and calcium-ion modification. The resulting material showed strong arsenic-adsorption performance [89]. Lignin-based arsenic-removal materials have been widely studied for water treatment but have received limited attention in soil remediation. A major limitation is that lignin generally requires complex modification before it can be used for soil treatment. Existing modification processes are relatively costly and may generate secondary pollution, reducing the economic competitiveness of lignin-based materials compared with other remediation amendments. Future research should therefore develop environmentally benign modification methods that maintain arsenic-removal performance while reducing production costs. Such efforts could support the development of efficient, low-cost, natural-polymer-based arsenic-removal materials and promote the practical application of lignin in the remediation of arsenic-contaminated soils [79,92].

## 4. Metal and Metal Oxide Functional Materials

Metal and metal oxide functional materials show considerable potential for the remediation of arsenic-contaminated soils because of their unique physicochemical properties. Table 2 summarizes the applications and arsenic-removal performance of these materials.

### 4.1. Nanoscale Zero-Valent Iron-Based Materials

Nanoscale zero-valent iron (nZVI) has attracted extensive interest for heavy-metal remediation because of its high reactivity and treatment efficiency. nZVI typically has a core–shell structure, with a Fe0 core surrounded by a shell composed of Fe (II)/Fe (III) oxides. This structure provides an electron source and surface sites for adsorption and complexation. It also retards rapid oxidation of the iron core and helps maintain material reactivity [17,93]. One study reported that nZVI treatment reduced plant arsenic uptake by 47% and decreased arsenic concentrations in soil pore water by 80% [94]. Xie et al. [95] investigated the simultaneous immobilization of arsenic and cadmium using sulfidated nano-zero-valent iron (S-nZVI). They found that sulfidation formed an FeSx shell on the nZVI surface. This shell inhibited excessive oxidation of nZVI and enhanced its selective adsorption of As(III). However, the direct application of nZVI for arsenic remediation in soils is often limited by particle aggregation and restricted transport. Therefore, nZVI generally requires stabilization or loading onto suitable supports [96,97].

With respect to support selection, Chen et al. [98] reviewed the synergistic degradation mechanisms of nZVI-modified biochar. They reported that biochar can serve as a support to disperse nZVI particles and enhance remediation performance through its abundant surface reactive sites. Studies of zeolite-supported nZVI (Z-nZVI) showed that zeolite reduced nZVI aggregation and promoted the uniform distribution of nZVI on the zeolite surface. In both acidic and alkaline soils, Z-nZVI converted arsenic into forms with low release potential [99]. Sepiolite-modified nZVI reduced the bioavailable arsenic fraction in soil by 45–80% under three water-management conditions [100]. When soils with different contamination levels were treated with poly(acrylic acid)-stabilized nZVI, As(V) and As(III) were adsorbed onto iron oxide surfaces. In addition, newly formed iron oxides generated during nZVI oxidation further promoted arsenic immobilization, resulting in effective stabilization [101]. Among the available support strategies, biochar-supported nZVI has considerable potential for engineering applications because biochar feedstocks are widely available and the preparation process is relatively simple. Nevertheless, the synergistic mechanisms between biochar and nZVI and their long-term stability require further investigation [102].

Despite these advantages, nZVI-based materials face several challenges in practical applications. The transport distance of nZVI particles in soil is limited, and their effects are generally confined to a few centimeters around the injection point. This limitation restricts the remediation efficiency of large contaminated sites. nZVI particles may also adversely affect soil microbial communities. The release of high concentrations of Fe^2+^ and substantial local pH fluctuations may inhibit sensitive microorganisms and alter microbial community composition [100]. In addition, nanoparticles may migrate downward through soil pore water, posing a potential risk to groundwater. Prolonged exposure to air and water also causes gradual oxidation and deactivation of nZVI. Its effective lifetime and long-term stability under field conditions have not yet been systematically evaluated [103].

### 4.2. Metal Oxide-Based Materials

In the remediation of arsenic-contaminated soils, metal oxides, particularly manganese and iron oxides, are widely used because of their high surface reactivity, redox activity, remediation efficiency, and environmental compatibility [17,41]. Iron- and manganese-based oxides generally show greater arsenic immobilization capacity than other metal oxides, including Al_2_O_3_, TiO_2_, and ZrO_2_. This advantage is mainly related to their distinct surface and redox properties. Iron oxide surfaces contain abundant reactive hydroxyl groups. Arsenate can undergo ligand exchange with these groups to form stable inner-sphere complexes [104,105]. In addition, the formation of inner-sphere As(V) complexes on iron oxide surfaces contributes to arsenic retention, while Fe(II)/Fe(III) redox cycling can promote transformations in arsenic speciation [106]. Manganese oxides provide a further advantage because they are among the strongest naturally occurring oxidants in the environment. Their Mn(IV)/Mn(III) redox couple can rapidly oxidize As(III) to As(V). In contrast, Ti(IV) and Zr(IV) generally occur in stable oxidation states and cannot support this redox-driven oxidation process [107,108]. Iron and manganese are abundant elements in the Earth’s crust, and their oxides occur naturally in soils. Therefore, iron- and manganese-based oxides generally have better environmental compatibility and lower ecological risk than synthetic Ti- and Zr-based materials.

The chemical properties of manganese oxides are closely related to their structures. MnO_6_ octahedra are the fundamental structural units of manganese oxides and are commonly linked through edge-sharing and corner-sharing arrangements to form tunnel or layered structures. Structural defects and vacancies within MnO_6_ octahedra can alter surface charge, whereas edge sites provide reactive sites for adsorption and oxidation reactions [107]. Studies have shown that manganese oxides can inhibit arsenic mobility in arsenic-contaminated paddy soils. Their application also significantly reduced arsenic concentrations in rice grains and straw [109]. Biogenic manganese oxides (BMOs), produced through Mn (II) oxidation by manganese-oxidizing bacteria, can significantly reduce bioavailable arsenic in soil, with a maximum reduction of 62.3%. BMOs also altered soil bacterial community composition and diversity, which may favor the long-term control of arsenic contamination [110]. However, the oxidative capacity of manganese-based materials is strongly influenced by soil environmental conditions. Variations in pH and temperature can affect oxidation kinetics by changing mineral surface charge and reaction energy barriers. Consequently, the performance of manganese oxides in field applications depends strongly on soil properties, particularly pH and redox potential [107].

Iron oxides also offer important advantages for arsenic remediation because their surface properties favor arsenic immobilization. Goethite, a representative iron oxide, has shown high efficiency for remediating arsenic-contaminated soils. At the lowest application rate of 0.2%, goethite nanoparticles reduced TCLP-extractable arsenic by 82.5% without affecting soil pH or electrical conductivity, indicating good environmental compatibility [111]. However, the long-term stability of iron-oxide-based materials remains a challenge. Iron oxides with different crystalline phases exhibit markedly different arsenic adsorption capacities. Although ferrihydrite is among the iron oxides with the highest arsenic adsorption capacity, it gradually transforms into hematite and goethite after 360 days of aging in soil, resulting in a loss of surface sites and a decrease in adsorption capacity [112]. The presence of silicate can inhibit this phase transformation and thereby delay the decline in ferrihydrite adsorption performance [113]. In reducing soil environments, such as paddy fields, the reductive dissolution of iron oxides is a major mechanism driving arsenic release. The reductive dissolution of Fe(III) minerals accounts for 23.3% of the temporal variation in porewater As concentrations, while Geobacteraceae and Clostridium are key microbial groups involved in Fe(III) reduction and arsenic release [114,115]. Therefore, the long-term remediation performance of iron-oxide-based materials should be evaluated by considering the redox conditions of the target soil and the aging behavior of the materials under those conditions.

### 4.3. Multimetal Composite Materials

Multimetal composite materials, including Ce-Mn, Zr-Mn, and Fe-Mn systems, have shown promising performance in arsenic remediation. Fe-Mn binary oxides are widely distributed in natural environments and have received increasing attention because of their high As(III) removal capacity and their important role in the biogeochemical cycling of arsenic [116]. McCann et al. [117] prepared an Fe-Mn binary oxide from a water-treatment residual. The material exhibited adsorption capacities of 70 mg/g for As(III) and 32 mg/g for As(V), indicating its potential as a sustainable and low-cost material for soil remediation. In another study, Si et al. [118] developed biogenic Fe-Mn binary oxides (BFMOs) using manganese-oxidizing bacteria. The BFMOs removed 87.05% of As(III) and 94.23% of As(V) within 7 d and retained high removal efficiencies after 28 d.

To address the performance loss caused by Fe-Mn oxide aggregation, a surfactant-modified Fe-Mn composite oxide (TFMO) was prepared. Compared with the unmodified Fe-Mn oxide, TFMO exposed more active sites and significantly reduced arsenic bioavailability and mobility [119]. Certain industrial solid wastes can also serve as raw materials for Fe-Mn stabilizers. For example, an Fe-Mn stabilizer prepared by mechanically mixing steel slag, ferrous sulfate, and bismuthinite reduced arsenic bioavailability through adsorption, complexation, and coprecipitation, achieving an immobilization efficiency of 85% after 90 d. Its low raw-material cost and simple preparation process indicate potential for large-scale remediation of mine-contaminated sites [120]. The limitations of multimetal composite materials include an incomplete understanding of the synergistic interactions among their metal components and the lack of standardized criteria for optimizing their composition and preparation processes. The potential leaching of metal ions may result in secondary pollution. Therefore, their long-term environmental behavior must be systematically assessed before large-scale application. Overall, metal- and metal oxide-based functional materials are among the most extensively studied material classes for remediating arsenic-contaminated soils and have considerable potential for practical application.

**Table 2:** Metal and metal oxide functional materials for arsenic-contaminated soil remediation.

Type	Representative Material	Preparation/Modification Method	Application Dosage	Application Site	Primary Immobilization Mechanism	Arsenic Immobilization Efficiency	Advantages	Ref.
Nanoscale zero-valent iron	Poly(acrylic acid)-stabilized nZVI	Liquid-phase reduction + polymer coating	2.5% (*w*/*w*)	Lightly and heavily contaminated areas	Surface complexation, coprecipitation, adsorption by newly formed iron oxides	TCLP-extractable As decreased by 70% (lightly contaminated) and 65% (heavily contaminated)	High reactivity; newly formed iron oxides continuously provide adsorption sites	[101]
	Sepiolite-modified nZVI (S-nZVI)	Support loading + liquid-phase reduction	3% and 5% (*w*/*w*)	Laboratory simulation	Surface complexation, coprecipitation	Soil available As decreased by 45–80%	Good dispersion on support; stable performance under different water management conditions	[100]
	NANOFER STAR nZVI	Commercial surface-stabilized nanoscale zero-valent iron	1 wt.%	As-rich mining area soil, sunflower pot experiment	Surface complexation, Formation of secondary Fe-As phases	Porewater As decreased by >80%; root As decreased by 47%; shoot As decreased by 24%	Commercially available with stable properties; significantly reduces As phytoavailability	[94]
	Magnetic biochar-supported nZVI (nZVI-MBC)	Chemical coprecipitation + liquid-phase reduction	2% (*w*/*w*)	Cd-As co-contaminated agricultural soil	Surface adsorption, complexation, coprecipitation, radical-mediated oxidation	Total As decreased by 13.7% (via magnetic separation); bioavailability decreased by 34.1%	Magnetic separation enables physical removal of heavy metals (reduces total content); simultaneously increases soil pH, CEC	[102]
Metal oxide-based	Natural manganese oxides	Direct use of natural minerals	Natural content	Arsenic-contaminated paddy soils	Oxidation, surface complexation;	As mobilization in high-Mn soil (ZP) was 10–20 times lower than other soils	Strong oxidizing capacity; naturally occurring; inhibits As release by retarding Eh decline	[109]
	Synthetic Mn oxide (hausmannite)	Alkaline oxidation synthesis	1200 mg Mn/kg soil	As-contaminated paddy soils	Oxidation, promotes Fe(II) oxidation to form neoformed Fe(III) (hydr)oxides for As adsorption	Porewater As(III) significantly decreased; rice grain As decreased by 30–40%	Significantly reduces As mobility and plant uptake; no Mn toxicity observed	[109]
	Biogenic manganese oxides (BMO)	Mn(II)-oxidizing bacteria bio-oxidation	0.013–0.063% (*w*/*w*)	Laboratory simulation	Oxidation, adsorption, coprecipitation	Bioavailable As decreased from 4.56 to 1.72–1.86 mg/kg (decreased by 62.3%)	Can regulate soil microbial communities, favorable for long-term As control	[110]
	Goethite nanoparticles	Chemical precipitation/hydrothermal method	0.2% (*w*/*w*)	Fertilizer plant As-contaminated soil	Surface complexation, ligand exchange	TCLP-extractable As decreased by 82.5% (0.2%); 99.8% (5%)	High efficiency at low dosage; does not affect soil pH or electrical conductivity	[111]
Multimetal composite	Fe-Mn binary oxide from water treatment residual	Direct use of industrial byproduct	10% (*w*/*w*)	Former industrial site As-contaminated soil	Adsorption, oxidation, coprecipitation	As(III) adsorption capacity 70 mg/g; As(V) 32 mg/g	Very low cost; high sustainability	[117]
	Biogenic Fe-Mn binary oxides (BFMO)	Mn-oxidizing bacteria + Fe coprecipitation	10% (*v*/*v*)	Laboratory-simulated As-contaminated soil	Oxidation, surface complexation, coprecipitation	As(III) removal 87.05%; As(V) 94.23% (7 d)	High efficiency; good stability over 28 days	[118]
	Surfactant-modified Fe-Mn oxide (TFMO)	Surfactant modification (CTAB modification)	5% (*w*/*w*)	Laboratory simulation	Oxidation, surface complexation	Bioavailability decreased by 85.8%; residual As fraction increased from 30.5–41.6% to 67.9–69.2%	Enhanced exposure of active sites; good dispersion	[119]
	Solid waste-based Fe-Mn stabilizer	Mechanical mixing	5% (*w*/*w*)	Mining area As-contaminated soil	Adsorption, complexation, coprecipitation	90-day immobilization efficiency: 85%	Ultra-low raw material cost; simple preparation; suitable for large-scale mining site remediation	[120]

## 5. Industrial Solid Waste-Based Functional Materials

Industrial solid wastes are by-products generated during industrial production, including steel slag, coal gangue, red mud, and carbide slag. These materials often possess hierarchical pore structures, high specific surface areas, and diverse reactive components. Such properties can facilitate arsenic speciation transformation and immobilization in contaminated soils. However, the direct use of industrial solid wastes may pose environmental risks. Heavy metals, alkali metals, and soluble salts present in these materials may be gradually released into the soil, causing secondary contamination. Therefore, the long-term stability and environmental safety of industrial-solid-waste-based functional materials are critical factors governing their engineering applications [121,122].

### 5.1. Steel Slag

Steel slag is a typical by-product of the iron and steel industry. Its mineral composition varies with processing conditions, but it generally contains abundant iron- and calcium-bearing oxides, supporting its beneficial reuse in environmental remediation [122]. Zero-valent iron (Fe^0^) and iron oxides in steel slag can undergo oxidation and hydrolysis to form amorphous iron oxyhydroxides (FeOOH). These iron phases can immobilize arsenate through surface complexation and precipitation, leading to the formation of Fe–As precipitates [123,124]. An experimental study showed that the addition of 0.5% steel slag achieved an arsenic immobilization efficiency of up to 89% in arsenic-contaminated soil from a mining area [125]. Another study reported that H^+^ released during Fe(II) hydrolysis promoted the oxidation of Fe^0^ on the steel slag surface to Fe(II). This process facilitated the formation of crystalline FeOOH and amorphous Fe(III) oxyhydroxides. In addition, H^+^ promoted the release of Ca^2+^ from steel slag, which subsequently reacted with arsenic to form Ca–As precipitates. Based on this mechanism, a composite material prepared from steel slag and ferrous sulfate reduced available arsenic in soil by 90.7% [124].

Combining steel slag with carbon-containing solid wastes can introduce surface functional groups, including C-O, C=O, and -COOR groups. These groups may generate microscale electric-field effects, promote Fe(II)/Fe(III) redox cycling, and facilitate the interfacial formation of amorphous iron oxides. A ternary steel slag (SS)-coal fly ash (CFA)-Fe(II) composite system reduced available arsenic by 59.8% in lightly contaminated soil and by 86.7% in highly contaminated soil [126]. Steel slag contains heavy metals, including Cr, Pb, and Zn, which may be gradually released under acidic conditions or prolonged leaching, posing a risk of secondary contamination to soil and groundwater [127]. In addition, the strong alkalinity of steel slag may cause substantial changes in soil pH, thereby affecting soil microbial communities and crop growth [128]. Although the short-term remediation performance of Fe/C composite systems has been demonstrated, their long-term stability and the co-release of heavy metals under realistic environmental conditions have not been systematically assessed [129]. Therefore, long-term monitoring of the leaching toxicity and ecotoxicity of steel-slag-based materials is required before their large-scale application, particularly to clarify how their performance changes across different soil types and climatic conditions [121,130].

In arsenic-contaminated paddy soils, bioavailable silicon released from steel slag can compete with arsenic for uptake sites in rice roots, thereby reducing arsenic accumulation in rice plants [131,132]. Studies of paddy-soil remediation have confirmed that steel slag significantly increased soil available silicon and reduced arsenic concentrations in rice grains by more than 30% on average. The suppressive effect persisted over time [133].

### 5.2. Bauxite Residue (Red Mud)

Bauxite residue (red mud) is a highly alkaline metallurgical solid waste generated during alumina refining. Its major mineral phases are dominated by Fe_2_O_3_, Al_2_O_3_, CaO, and SiO_2_. Its porous structure and relatively high specific surface area provide adsorption sites for arsenic immobilization in soil through surface complexation involving hydroxyl groups and ion exchange [134,135]. The high alkalinity of bauxite residue has both beneficial and adverse effects. A high pH may promote arsenate adsorption and precipitation; however, the release of soluble alkali metals and heavy metals from bauxite residue may cause soil salinization and alkalinization, as well as secondary contamination. At high application rates, bauxite residue may induce genotoxic and cytotoxic effects in organisms [136]. In addition, its fine particles are prone to dust generation, which may adversely affect local air quality [137]. To reduce these environmental risks, modification strategies include acid neutralization, high-temperature calcination, and co-stabilization with calcium- or silicon-containing materials. For example, Ca^2+^/Cl^−^ dual-ion neutralization reduced the pH from 11.0 to 8.9 and decreased As leaching to below the applicable environmental limit [138]. The addition of phosphogypsum increased the As adsorption capacity of bauxite residue by 3.5-fold [139]. Moreover, RMPG, comprising 75% bauxite residue and 25% phosphogypsum, reduced As concentrations in plant stems by 93% [140]. However, these modification methods increase treatment costs; therefore, a balance between remediation efficiency and economic feasibility is required.

### 5.3. Magnesium Slag and Manganese Slag

Magnesium slag and manganese slag, both by-products of metal smelting, have also shown potential for arsenic immobilization in contaminated soils. Application of magnesium slag reduced soil available arsenic by 49.1% relative to the control and decreased total arsenic concentrations in rice grains by 21.8%. These results indicate that magnesium slag can inhibit arsenic transfer from soil to rice plants [141]. In another study, a pyrite-modified electrolytic manganese slag-based remediation material (M-EMS) was prepared [104]. M-EMS decreased the concentration of toxicity characteristic leaching procedure (TCLP)-leachable arsenic from 657.2 to 319.8 μg L^−1^ [142]. Manganese residues pose substantial environmental risks. Electrolytic manganese residue (EMR) contains relatively high concentrations of soluble Mn^2+^ and NH_4_^+^-N, which may be released into nearby water bodies and soils during rainfall leaching, resulting in secondary manganese contamination and soil acidification [143]. Pyrite-modified M-EMS has been shown to effectively immobilize arsenic; however, the stability of Mn^2+^ in the modified residue has not been thoroughly evaluated [142]. Magnesium slag contains free CaO and MgO, which can hydrate upon contact with water and cause volume expansion, potentially compromising its structural stability in soil and the long-term durability of its remediation performance [144]. In addition, the co-release of associated heavy metals, including Pb, Zn, and Cu, from magnesium and manganese residues under acidic conditions warrants attention. The application of magnesium-slag- and manganese-residue-based materials therefore requires effective control measures tailored to their target contaminants, together with long-term assessments of their ecological risks.

### 5.4. Coal Gangue

Coal gangue is a solid waste generated during coal mining and coal washing. Owing to its abundant surface reactive sites and relatively high specific surface area, it is a potential precursor for low-cost and efficient soil amendments [145,146]. However, unmodified coal gangue has limited effectiveness for the remediation of arsenic-contaminated soils. Therefore, iron-based modification has been used to produce iron oxyhydroxide-modified coal gangue (CG-FeOH), iron hydroxy sulfate-modified coal gangue (CG-FeOS), and a ferrous sulfate-modified coal gangue-based magnetic porous material (MPCG). These modified coal gangue materials can decrease arsenic bioavailability through adsorption, redox reactions, complexation, and coprecipitation, showing considerable potential for soil remediation [147,148]. The environmental risks of coal gangue mainly arise from its heavy-metal content. The leaching concentrations of As, Co, Cu, Mn, Ni, Pb, and Zn may exceed the relevant water-quality standards by 1.1–46.6 times [149,150]. Existing studies have mainly relied on laboratory simulations [147,148]; therefore, the long-term stability and environmental safety of coal-gangue-based materials in field remediation require further evaluation. The resource utilization of coal gangue requires effective measures to immobilize heavy metals, particularly in regions with high rainfall or shallow groundwater tables.

## 6. Clay Mineral-Based Functional Materials

Clay minerals are naturally occurring hydrous aluminosilicates with layered structures. In soil science, the clay fraction is commonly defined as particles with an equivalent diameter of <2 μm. Because of their high specific surface area, cation-exchange capacity, and structural diversity, clay minerals are widely used as natural adsorbents for environmental contaminants. Their basic structures comprise silicon–oxygen tetrahedral sheets and aluminium–oxygen octahedral sheets. Based on the arrangement of these sheets, clay minerals can be classified as 1:1 types, such as kaolinite and halloysite, and 2:1 types, such as montmorillonite and sepiolite [151,152].

Kaolinite is a 1:1 layered aluminosilicate mineral. It can adsorb arsenic in soil and alter arsenic speciation through pH regulation, thereby decreasing arsenic bioavailability. Aluminium-containing sites in the mineral structure may form stable complexes with arsenic and promote its in situ immobilization [153]. Metakaolin is produced by calcining kaolin at high temperature and consists mainly of highly reactive, amorphous aluminosilicates. Its surface contains abundant reactive sites that can exchange ligands with arsenate ions, forming inner-sphere complexes. Metakaolin is produced by the thermal calcination of kaolinite and generally exhibits higher reactivity than the parent mineral. A metakaolin–limestone stabilization material reduced arsenic leachability by 96.2% [154], whereas a metakaolin–red mud material reduced the arsenic leaching concentration to below the relevant safety threshold [155].

Montmorillonite is a 2:1 layered aluminosilicate mineral with a permanent negative surface charge [156,157]. For arsenic immobilization, its interfacial properties and reactivity generally require optimization through physical [158], inorganic [159], or organic modification [160]. Fe(III)-modified montmorillonite can be prepared by treating montmorillonite with FeCl_3_ solution. This material achieved an As(III) oxidation rate of approximately 90% and substantially decreased arsenic mobility and toxicity [161]. Similarly, goethite- and ferrihydrite-modified montmorillonites showed improved arsenic removal performance [162,163]. Dodecyltrimethylammonium (DTMA)-modified bentonite (D-Bents) was also prepared for arsenic stabilization. Soil remediation experiments showed that D-Bents reduced arsenic leachability by up to 39.0% [164]. Bentonite is a natural clay material composed predominantly of montmorillonite and commonly contains accessory minerals, including quartz and kaolinite [165]. Zirconium-loaded, lanthanum-modified bentonite (Zr-LMB), developed by exploiting the strong affinity of zirconium oxides for arsenic, reduced arsenic release from sediments by up to 74.4% [166].

Natural clay minerals typically have negatively charged surfaces. Electrostatic repulsion from anionic arsenic species limits their direct adsorption, so metal loading or organic modification is often required to reverse or screen the surface charge [167,168]. Although the interlayer regions of clay minerals generally have a high cation-exchange capacity, their anion-exchange capacity is limited. Consequently, arsenic adsorption depends more on surface complexation than on ion exchange [169]. In terms of long-term stability, the gradual release of metal ions from metal-loaded clay minerals may continuously provide reactive sites but may also cause secondary contamination. Their long-term environmental behavior remains unclear [170]. Metal-loaded clay minerals can be regenerated by acid washing or desorption with chelating agents. However, these treatments may cause the dissolution of interlayer metals and structural damage. Research on the regeneration and reuse of clay minerals in soil remains limited.

## 7. Molecular Sieve-Based Functional Materials

Molecular sieves are microporous crystalline materials with regular pore structures. According to their origin, they can be divided into natural zeolites and synthetic molecular sieves. These materials can immobilize arsenic through ion exchange, adsorption, and precipitation [171]. In natural zeolites, isomorphous substitution of SiO_4_ tetrahedra by AlO_4_ tetrahedra produces a negatively charged framework. Consequently, natural zeolites generally exhibit limited adsorption of arsenic oxyanions [172]. Current modification strategies mainly include surfactant treatment, organic functionalization, and metal loading [173,174]. Surfactant modification reverses the zeolite surface charge from negative to positive by forming a positively charged organic cation layer, such as hexadecyltrimethylammonium, on the zeolite surface. This modification enhances the electrostatic attraction of arsenate anions [175]. Metal loading immobilizes arsenic by introducing metal oxides, such as iron and zirconium oxides, which promote ligand exchange and surface complexation with arsenic species. Li et al. [99] prepared a zeolite-based composite by loading nanoscale zero-valent iron (nZVI) onto zeolite. Tests using agricultural soil showed that this composite reduced arsenic mobility and bioavailability. Although natural zeolite is inexpensive, its modification requires the addition of surfactants or other chemical reagents. The ecotoxicity of residual modifiers therefore requires evaluation. In the field of synthetic molecular sieves, Yang et al. [176] converted natural soil into an Fe_2_O_3_-CAN-type zeolite composite. Owing to its anion-exchange capacity and strong adsorption capacity, the composite reduced arsenic accumulation in plants by 48.1–67.0%. However, metals such as Fe and Zr introduced into metal-loaded molecular sieves may leach under acidic conditions or during prolonged leaching, potentially causing secondary contamination. Although the “soil-for-soil” strategy is conceptually innovative, the complex composition of natural soil may affect the process stability and product consistency of its conversion into functional molecular sieves. These aspects require systematic investigation [177].

## 8. Conclusions and Perspectives

A systematic comparison indicates that functional materials differ in their advantages and suitability for the remediation of arsenic-contaminated soils (Table 3), whereas Table 4 compares six major arsenic immobilization mechanisms. Iron- and manganese-based materials are among the most effective arsenic immobilization agents because they can combine ligand exchange, coprecipitation, redox transformation, and electrostatic attraction. These materials show high immobilization efficiencies for both As(III) and As(V) and are applicable to a broad range of arsenic-contaminated sites. Biochar-based materials are inexpensive and readily modified. When combined with iron and manganese oxides, their remediation performance can equal or exceed that of single metal-oxide materials, offering a practical and cost-effective option. Materials derived from industrial solid wastes have low feedstock costs and support the principle of waste valorization. They therefore offer economic advantages for large-scale remediation of mining areas and agricultural soils. Natural polymeric materials generally show limited direct arsenic adsorption but provide versatile support matrices for functional modification. Clay minerals and molecular sieves are abundant and environmentally compatible; however, their adsorption capacities are generally lower than those of metal oxides. They are therefore more suitable as auxiliary components in composite materials.

Despite substantial progress in the use of functional materials for arsenic-contaminated soil remediation, several scientific questions and technical challenges remain.

(1) Uncertain long-term effectiveness of remediation materials. Most studies have evaluated remediation performance over only weeks or months. However, soil pH fluctuations, wetting–drying cycles, microbial activity, and changes in soil organic matter can alter material surface properties and arsenic immobilization over time. Iron-based materials may promote arsenic remobilization under periodically reducing conditions, whereas organically modified materials may lose effectiveness because of modifier degradation. Systematic monitoring data over periods exceeding three years remain scarce.
(2) Regeneration and reuse of remediation materials. Most functional materials are currently used as single-use amendments, and economically viable regeneration approaches are limited. Magnetic materials can be recovered to some extent by magnetic separation, but their adsorption performance often declines after repeated regeneration cycles. Efficient in situ regeneration methods and low-cost material recovery strategies are needed to reduce the life-cycle cost of soil remediation.
(3) Insufficient environmental safety assessment. The environmental risks of remediation materials have not been fully evaluated. These risks include the ecotoxicity of nanomaterials to soil microorganisms, plant roots, and soil fauna; the leaching of metal ions from metal oxides; and secondary contamination caused by potentially toxic elements in industrial solid wastes. Remediation materials should immobilize arsenic without introducing additional environmental risks. Therefore, systematic environmental safety assessment should be incorporated into the early stages of material development.
(4) Limited translation from laboratory studies to field application. Most studies have been conducted under controlled laboratory conditions or in small-scale pot experiments. The effects of soil type, water content, organic matter content, and competing anions, such as phosphate and silicate, on remediation performance have not been adequately assessed. Pilot-scale and field-scale validation are still limited, which restricts the transferability of reported performance to complex contaminated sites. To address these challenges, the following research priorities are proposed.
(5) Development of multifunctional composite materials. Future material design should move beyond arsenic immobilization alone toward the integrated functions of arsenic immobilization, soil improvement, and ecological restoration. For example, composites containing iron and manganese oxides, biochar, and slow-release nutrient sources may immobilize arsenic while improving soil structure and fertility and facilitating the recovery of soil microbial communities.
(6) Hybrid composites and synergistic remediation strategies. A single material often cannot meet the remediation requirements of complex contaminated sites. Future research should therefore focus on the rational combination of different material types. Examples include optimizing the ratio of manganese-based and iron-based materials and refining the loading of nanoscale zero-valent iron onto biochar. In such composites, biochar can provide a porous carbon matrix, whereas nanoscale zero-valent iron provides reactive sites. Functional complementarity among components may thereby improve overall remediation performance.
(7) Pilot-scale and field-scale validation. Long-term pilot-scale and field trials are urgently needed at different arsenic-contaminated sites, including mining areas, agricultural soils, and industrial brownfields. These trials should systematically evaluate remediation effectiveness, long-term stability, and environmental safety under realistic conditions.
(8) Green and low-cost preparation processes. The transition of functional materials from laboratory research to engineering application requires lower production costs and reduced environmental impacts. Priority should be given to remediation materials produced from low-cost feedstocks, including agricultural residues and industrial solid wastes. Simplified processes, such as one-step synthesis and mechanochemical methods, should also be explored. In addition, life-cycle cost assessments are needed to evaluate their economic feasibility.
(9) Material regeneration and recycling technologies. Regeneration methods should be developed for different types of remediation materials. Quantitative relationships between regeneration efficiency and the number of reuse cycles should also be established. Such information would support material selection and replacement schedules in engineering applications.

In summary, future research on functional materials for arsenic-contaminated soil remediation should prioritize innovation, sustainability, effectiveness, and practical applicability. Material design should emphasize multifunctional integration and synergistic effects, while research methods should incorporate field validation and standardized evaluation. Engineering applications should balance cost control with environmental safety. Multidisciplinary research efforts are needed to advance functional materials from laboratory studies to large-scale implementation and to provide sustainable solutions for soil ecological security and human health.

**Table 3:** Comprehensive comparison of six major categories of functional materials for arsenic-contaminated soil remediation.

Material Category	Preparation Method	Immobilization Mechanism	Efficiency	Advantages	Limitations	Cost	Technology Readiness	Ref.
Biochar-based	Pyrolysis of biomass (300–900 °C) with metal salt loading	Surface complexation, ligand exchange, redox transformation, coprecipitation	High (arsenic release decreased by 77%; residual As fraction increased by 161%)	Wide feedstock availability; carbon sequestration; soil amelioration; readily functionalizable	Performance depends on feedstock and pyrolysis conditions	Low	Field application	[38,39,43,46]
Graphene- based	Chemical synthesis (oxidation–reduction) with metal oxide compositing	Surface complexation, electrostatic attraction, π–π interactions	High (removal efficiency 84.8–98.5%)	Large specific surface area; highly designable; high adsorption capacity	Very high cost; ecological risks; high mobility	Very high	Laboratory	[54,57,58,59]
Hydrochar- based	Hydrothermal carbonization (180–260 °C) with metal salt loading	Surface complexation, electrostatic interactions	Moderate–high (As leaching decreased by >94%; immobilization efficiency 71.62%)	Direct processing of wet feedstocks; rich functional groups; relatively low energy consumption	Low specific surface area; insufficient long-term stability; process water requires treatment	Moderate	Pilot	[63,64]
Chitosan-based	Deacetylation of chitin, crosslinking, metal loading	Electrostatic attraction (protonated amino groups), complexation	Moderate (immobilization efficiency 73.98%; BCC decreased As by 85%)	Abundant amino/hydroxyl groups; biodegradable; numerous modification sites	Dissolution under acidic conditions; performance decline after repeated regeneration; high cost	Low–moderate	Laboratory to pilot	[72,73,76]
Cellulose-based	Extraction from plant biomass, crosslinking, metal/metal oxide loading	Complexation (as support material)	Moderate (TCLP-extractable As removal 65%; CMC-stabilized As immobilization >99.9%)	Widely available; low cost; good mechanical strength; mature processing technology	Limited direct electrostatic attraction for As oxyanions; requires functional component loading	Low	Pilot	[83,84]
Lignin-based	Extraction from biomass, doping/grafting/crosslinking modification	Complexation (active sites introduced after modification)	Moderate (significantly enhanced adsorption after modification)	Rigid aromatic backbone; good chemical stability; rich functional groups	Negatively charged surface repels As oxyanions; requires complex modification	Low–moderate	Laboratory	[89,91]
nZVI-based	Liquid-phase reduction (Fe(III) to Fe(0)) with polymer/support stabilization	Surface complexation, coprecipitation, adsorption by newly formed iron oxides	High (TCLP-extractable As decreased by 70% or 65%; plant As uptake decreased by 47%)	High reactivity; strong electron-donating capacity	Particle aggregation; limited transport (centimeter-scale)	Moderate	Pilot to field	[94,100,101,102]
Mn/Fe oxide-based	Natural minerals, chemical precipitation, hydrothermal synthesis	Oxidation (As(III) to As(V)), surface complexation, coprecipitation	High (goethite: TCLP-extractable As decreased by 82.5% at 0.2% dosage)	Strong oxidation capacity; naturally occurring; environmentally compatible	Performance depends on soil conditions, particularly pH and redox potential; long-term stability uncertain	Moderate–high	Pilot to field	[109,110,111]
Multimetal composite	Coprecipitation, hydrothermal synthesis, solid waste mechanical mixing	Redox transformation, surface complexation, coprecipitation	High (As(III) adsorption capacity 70 mg/g; As(V) adsorption capacity 32 mg/g; immobilization efficiency 85%)	Synergistic effects; cost-controllable; enables solid waste resource utilization	Unclear synergistic mechanisms among components; metal leaching risk	Moderate	Pilot to field	[116,117,118,119,120]
Steel slag-based	Mechanical crushing, screening, activation (physical or chemical)	Complexation, precipitation, ion exchange	Moderate–high (immobilization efficiency 89%; As leaching decreased by 90.7%)	Rich in Fe/Ca oxides; widely available	Heavy metal (Cr/Pb) leaching risk; strong alkalinity	Very low	Pilot to field	[124,125,127,129]
Red mud-based	Mechanical mixing, acid neutralization, thermal activation	Surface hydroxyl complexation, ion exchange	Moderate–high (stem As decreased by 93%; adsorption capacity increased 3.5-fold)	Rich in Fe/Al oxides; hierarchical pore structure	High alkalinity; V/Cr leaching risk; fine particle dust	Very high	Pilot to field	[134,135,136,137,138,139,140]
Mg slag/Mn slag-based	Mechanical activation, pyrite/sulfate modification	Adsorption, coprecipitation	Moderate (TCLP-extractable As decreased from 657.2 to 319.8 μg/L)	Waste-to-resource; low cost	Mn^2+^/NH_4_^+^-N leaching risk; volume expansion	High	Laboratory to pilot	[141,142,143,144]
Coal gangue-based	Iron salt loading, thermal modification	Adsorption, redox reactions, complexation, coprecipitation	Moderate (available As decreased by 13.46–54.37%)	Abundant surface active sites; magnetically separable	Heavy metal (Pb/Zn/Cu) leaching; long-term stability and environmental safety uncertain	High	Laboratory	[145,146,147,148,149,150]
Clay minerals-based	High-temperature calcination (kaolin to metakaolin), ion exchange, metal loading	Ligand exchange (Al-OH sites), surface complexation	Moderate–high (As leaching decreased by 96.2%; As leaching decreased by 39–74.4%)	Abundant in nature; environmentally friendly; high cation exchange capacity	Electrostatic repulsion of As oxyanions; requires modification to enhance adsorption	High	Pilot	[155,156,165,167,168,169,170]
Zeolite-based/ synthetic molecular sieves	Ion exchange, metal loading, soil transformation, high-temperature synthesis	Ion exchange, complexation, electrostatic attraction, anion exchange	Moderate (plant As accumulation decreased by 48.1–67.0%)	Regular pore structure; highly designable; “soil-for-soil” innovative strategy	Natural zeolites have low affinity for As oxyanions; high synthesis energy consumption	Moderate	Laboratory	[99,173,176,177]

**Table 4:** Comparison of six major arsenic immobilization mechanisms.

Mechanism	Essence	Binding Strength	Reversibility	Environmental Sensitivity	Dominant Material Types	Typical Example	Ref.
Surface complexation	Arsenic oxyanions form coordination bonds with metal hydroxyl groups (=FeOH, =MnOH, =AlOH, etc.) on material surfaces	High	Irreversible (inner-sphere)	Significantly influenced by pH; affected by soil redox conditions	Biochar-based, nZVI-based, Mn/Fe oxide-based, multimetal composite, clay minerals-based	Inner-sphere bidentate binuclear FeOOH-As(V) complexes	[104,105,106]
Ligand exchange	Arsenic oxyanions replace surface hydroxyl groups (-OH) or water molecules on material surfaces	High	Irreversible	Consistent with surface complexation; represents different perspective of the same reaction	Mn/Fe oxide-based, clay minerals-based	Arsenate replacing surface -OH on ferrihydrite	[104,105,154]
Coprecipitation	As combines with cations such as Fe^3+^ and Ca^2+^ to form sparingly soluble arsenate precipitates	Very high	Irreversible	Low (precipitates are stable)	nZVI-based, Mn/Fe oxide-based, multimetal composite, steel slag-based	FeAsO_4_ and Ca–As precipitates	[120,124]
Redox transformation	Variable-valence metals (Mn, Fe) oxidize As(III) to As(V)	Not applicable	Not applicable	Transformation efficiency affected by Eh/pH conditions	Mn/Fe oxide-based, multimetal composite	MnO_2_ oxidizing As(III) to As(V)	[107,108]
Electrostatic interaction	Coulombic attraction between positively charged surfaces (-NH_3_^+^, =FeOH_2_^+^) and negatively charged arsenic oxyanions	Low–moderate	Reversible (easily affected by ionic strength)	Very high (significantly influenced by pH and ionic strength)	Chitosan-based, graphene-based, zeolite-based/synthetic molecular sieves	HDTMA^+^-modified zeolite adsorbing H_2_AsO_4_^−^; protonated amino groups on chitosan adsorbing arsenate; electrostatic repulsion between nGOx surface and arsenate	[56,72,175]
Ion exchange	Arsenate exchanges with anions (Cl^−^, OH^−^, SO_4_^2−^, etc.) on material surfaces or interlayers	Low	Reversible (affected by ionic strength)	High (significantly influenced by coexisting competing anions)	Clay minerals-based, zeolite-based/synthetic molecular sieves, hydrochar-based	Arsenate exchange with surface anions on surfactant-modified zeolite; ion exchange in modified bentonite	[164,169,171,175]

## Data Availability

No new data were created or analyzed in this study. Data sharing is not applicable to this article.

## References

[1] Sevak P., Pushkar B. (2024). Arsenic Pollution Cycle, Toxicity and Sustainable Remediation Technologies: A Comprehensive Review and Bibliometric Analysis. J. Environ. Manag..

[2] Bundschuh J., Niazi N.K., Alam M.A., Berg M., Herath I., Tomaszewska B., Maity J.P., Ok Y.S. (2022). Global Arsenic Dilemma and Sustainability. J. Hazard. Mater..

[3] Okoye E.A., Bocca B., Ruggieri F., Ezejiofor A.N., Nwaogazie I.L., Frazzoli C., Orisakwe O.E. (2022). Arsenic and Toxic Metals in Meat and Fish Consumed in Niger Delta, Nigeria: Employing the Margin of Exposure Approach in Human Health Risk Assessment. Food Chem. Toxicol..

[4] Patel K.S., Pandey P.K., Martín-Ramos P., Corns W.T., Varol S., Bhattacharya P., Zhu Y. (2023). A Review on Arsenic in the Environment: Contamination, Mobility, Sources, and Exposure. RSC Adv..

[5] Pan Z., Gong T., Liang P. (2024). Heavy Metal Exposure and Cardiovascular Disease. Circ. Res..

[6] Fatoki J.O., Badmus J.A. (2022). Arsenic as an Environmental and Human Health Antagonist: A Review of Its Toxicity and Disease Initiation. J. Hazard. Mater. Adv..

[7] Vainio H., Heseltine E., Wilbourn J. (1994). Priorities for Future IARC Monographs on the Evaluation of Carcinogenic Risks to Humans. Environ. Health Perspect..

[8] Meharg A.A., Meharg C. (2021). The Pedosphere as a Sink, Source, and Record of Anthropogenic and Natural Arsenic Atmospheric Deposition. Environ. Sci. Technol..

[9] Xu W., Jin Y., Zeng G. (2024). Introduction of Heavy Metals Contamination in the Water and Soil: A Review on Source, Toxicity and Remediation Methods. Green Chem. Lett. Rev..

[10] Hou D., Jia X., Wang L., McGrath S.P., Zhu Y.-G., Hu Q., Zhao F.-J., Bank M.S., O’Connor D., Nriagu J. (2025). Global Soil Pollution by Toxic Metals Threatens Agriculture and Human Health. Science.

[11] Podgorski J., Berg M. (2020). Global Threat of Arsenic in Groundwater. Science.

[12] Zhang S., Zhang J., Niu L., Chen Q., Zhou Q., Xiao N., Man J., Ma J., Wei C., Zhang S. (2024). Escalating Arsenic Contamination throughout Chinese Soils. Nat. Sustain..

[13] Alka S., Shahir S., Ibrahim N., Ndejiko M.J., Vo D.-V.N., Manan F.A. (2021). Arsenic Removal Technologies and Future Trends: A Mini Review. J. Clean. Prod..

[14] Lanjwani M.F., Tuzen M., Khuhawar M.Y., Sarı A., Altunay N., Saleh T.A. (2026). Emerging Technologies and Advanced Nanomaterials for Arsenic Remediation in Water and Environmental Systems: A Comprehensive Review. Environ. Surf. Interfaces.

[15] Murthy M.K., Khandayataray P., Mohanty C.S., Pattanayak R. (2024). A Review on Arsenic Pollution, Toxicity, Health Risks, and Management Strategies Using Nanoremediation Approaches. Rev. Environ. Health.

[16] Wang A., Kong L., Wang J., Zhang Z., Fan H. (2026). Advancement of Organic/Inorganic Stabilization Materials for Remediation of Chromium and Arsenic Pollution: A Review. J. Water Process Eng..

[17] Tian Y., Qu G., Xu R., Liu X., Jin C. (2024). Iron-Based Materials for Immobilization of Heavy Metals in Contaminated Soils: A Critical Review. J. Environ. Chem. Eng..

[18] Simić M., Koprivica M., Dimitrijević J., Ercegović M., Anđić D., Fiol N., Petrović J. (2026). Valorization of Corn Processing Waste as Adsorbents for Soil and Water Remediation: A Systematic and Comparative Review of Native Biomass, Hydrochar, and Biochar. Processes.

[19] Vithanage M., Herath I., Joseph S., Bundschuh J., Bolan N., Ok Y.S., Kirkham M.B., Rinklebe J. (2017). Interaction of Arsenic with Biochar in Soil and Water: A Critical Review. Carbon.

[20] Gopinath K.P., Vo D.-V.N., Gnana Prakash D., Adithya Joseph A., Viswanathan S., Arun J. (2021). Environmental Applications of Carbon-Based Materials: A Review. Environ. Chem. Lett..

[21] Hakeem K.R., Alharby H.F., Bamagoos A.A.M., Pirzadah T.B. (2022). Biochar Promotes Arsenic (As) Immobilization in Contaminated Soils and Alleviates the As-Toxicity in Soybean. Chemosphere.

[22] Shi X., Yang W., Li J., Yao Z. (2024). The Application of Biochar as Heavy Metals Adsorbent: The Preparation, Mechanism, and Perspectives. Int. J. Environ. Res..

[23] Zhang K., Yi Y., Fang Z. (2023). Remediation of Cadmium or Arsenic Contaminated Water and Soil by Modified Biochar: A Review. Chemosphere.

[24] Rajapaksha A.U., Chen S.S., Tsang D.C.W., Zhang M., Vithanage M., Mandal S., Gao B., Bolan N.S., Ok Y.S. (2016). Engineered/Designer Biochar for Contaminant Removal/Immobilization from Soil and Water: Potential and Implication of Biochar Modification. Chemosphere.

[25] Wongrod S., Simon S., Van Hullebusch E.D., Lens P.N.L., Guibaud G. (2018). Changes of Sewage Sludge Digestate-Derived Biochar Properties after Chemical Treatments and Influence on As(III and V) and Cd(II) Sorption. Int. Biodeterior. Biodegrad..

[26] Amin M., Ahmad M., Farooqi A., Hussain Q., Ahmad M., Al-Wabel M.I., Saleem H. (2020). Arsenic Release in Contaminated Soil Amended with Unmodified and Modified Biochars Derived from Sawdust and Rice Husk. J. Soils Sediments.

[27] Cha J.S., Park S.H., Jung S.-C., Ryu C., Jeon J.-K., Shin M.-C., Park Y.-K. (2016). Production and Utilization of Biochar: A Review. J. Ind. Eng. Chem..

[28] Frišták V., Moreno-Jimenéz E., Fresno T., Diaz E. (2018). Effect of Physical and Chemical Activation on Arsenic Sorption Separation by Grape Seeds-Derived Biochar. Separations.

[29] Kumar M., Xiong X., Wan Z., Sun Y., Tsang D.C.W., Gupta J., Gao B., Cao X., Tang J., Ok Y.S. (2020). Ball Milling as a Mechanochemical Technology for Fabrication of Novel Biochar Nanomaterials. Bioresour. Technol..

[30] Amusat S.O., Kebede T.G., Dube S., Nindi M.M. (2021). Ball-Milling Synthesis of Biochar and Biochar–Based Nanocomposites and Prospects for Removal of Emerging Contaminants: A Review. J. Water Process Eng..

[31] Peter A., Chabot B., Loranger E. (2021). Enhanced Activation of Ultrasonic Pre-Treated Softwood Biochar for Efficient Heavy Metal Removal from Water. J. Environ. Manag..

[32] Luo M., Lin H., He Y., Li B., Dong Y., Wang L. (2019). Efficient Simultaneous Removal of Cadmium and Arsenic in Aqueous Solution by Titanium-Modified Ultrasonic Biochar. Bioresour. Technol..

[33] Yang X., Wan Y., Zheng Y., He F., Yu Z., Huang J., Wang H., Ok Y.S., Jiang Y., Gao B. (2019). Surface Functional Groups of Carbon-Based Adsorbents and Their Roles in the Removal of Heavy Metals from Aqueous Solutions: A Critical Review. Chem. Eng. J..

[34] Hussain M., Imran M., Abbas G., Shahid M., Iqbal M., Naeem M.A., Murtaza B., Amjad M., Shah N.S., Ul Haq Khan Z. (2020). A New Biochar from Cotton Stalks for As (V) Removal from Aqueous Solutions: Its Improvement with H3PO4 and KOH. Environ. Geochem. Health.

[35] Wang C., Qiao J., Yuan J., Tang Z., Chu T., Lin R., Wen H., Zheng C., Chen H., Xie H. (2024). Novel Chitosan-Modified Biochar Prepared from a Chinese Herb Residue for Multiple Heavy Metals Removal: Characterization, Performance and Mechanism. Bioresour. Technol..

[36] Li B., Gong J., Fang J., Zheng Z., Fan W. (2021). Cysteine Chemical Modification for Surface Regulation of Biochar and Its Application for Polymetallic Adsorption from Aqueous Solutions. Environ. Sci. Pollut. Res..

[37] Chen Y., Lin Q., Wen X., He J., Luo H., Zhong Q., Wu L., Li J. (2023). Simultaneous Adsorption of As(III) and Pb(II) by the Iron-Sulfur Codoped Biochar Composite: Competitive and Synergistic Effects. J. Environ. Sci..

[38] Wan X., Li C., Parikh S.J. (2020). Simultaneous Removal of Arsenic, Cadmium, and Lead from Soil by Iron-Modified Magnetic Biochar. Environ. Pollut..

[39] Song P., Xu H., Sun S., Xiong W., Yang Z. (2022). Remediation of Arsenic-Spiked Soil by Biochar-Loaded Nanoscale Zero-Valent Iron: Performance, Mechanism, and Microbial Response. J. Clean. Prod..

[40] Liang M., Lu L., Zhang Q., Li J., Qiao M., Wu Z. (2024). Time Scale Effect of Magnetic Ferric Oxide Modified Biochar In-Situ Remediation of Arsenic-Contaminated Paddy Soil. Environ. Technol. Innov..

[41] Younas M., Bacha A.U.R., Khan K., Nabi I., Ullah Z., Humayun M., Hou J. (2024). Application of Manganese Oxide-Based Materials for Arsenic Removal: A Review. Sci. Total Environ..

[42] Lin L., Li Z., Liu X., Qiu W., Song Z. (2019). Effects of Fe-Mn Modified Biochar Composite Treatment on the Properties of As-Polluted Paddy Soil. Environ. Pollut..

[43] Cuong D.V., Wu P.-C., Chen L.-I., Hou C.-H. (2021). Active MnO2/Biochar Composite for Efficient As(III) Removal: Insight into the Mechanisms of Redox Transformation and Adsorption. Water Res..

[44] Yu Z., Qiu W., Wang F., Lei M., Wang D., Song Z. (2017). Effects of Manganese Oxide-Modified Biochar Composites on Arsenic Speciation and Accumulation in an Indica Rice (*Oryza sativa* L.) Cultivar. Chemosphere.

[45] Yang Z., Zeng G., Liu L., He F., Arinzechi C., Liao Q., Yang W., Si M. (2023). Simultaneous Immobilization of Lead, Cadmium and Arsenic in Soil by Iron-Manganese Modified Biochar. Front. Environ. Sci..

[46] Zhang L., Hu J., Li C., Chen Y., Zheng L., Ding D., Shan S. (2023). Synergistic Mechanism of Iron Manganese Supported Biochar for Arsenic Remediation and Enzyme Activity in Contaminated Soil. J. Environ. Manag..

[47] Zhou J., Liu Y., Li B., Huang W., Qin J., Li H., Chen G. (2022). Hydrous Zirconium Oxide Modified Biochar for in Situ Remediation of Arsenic Contaminated Agricultural Soil. J. Environ. Chem. Eng..

[48] Zhang G., Liu X., Gao M., Song Z. (2020). Effect of Fe–Mn–Ce Modified Biochar Composite on Microbial Diversity and Properties of Arsenic-Contaminated Paddy Soils. Chemosphere.

[49] Chen Z., Wang Y., Xia D., Jiang X., Fu D., Shen L., Wang H., Li Q.B. (2016). Enhanced Bioreduction of Iron and Arsenic in Sediment by Biochar Amendment Influencing Microbial Community Composition and Dissolved Organic Matter Content and Composition. J. Hazard. Mater..

[50] Wu C., Zhi D., Yao B., Zhou Y., Yang Y., Zhou Y. (2022). Immobilization of Microbes on Biochar for Water and Soil Remediation: A Review. Environ. Res..

[51] Ji X., Wan J., Wang X., Peng C., Wang G., Liang W., Zhang W. (2022). Mixed Bacteria-Loaded Biochar for the Immobilization of Arsenic, Lead, and Cadmium in a Polluted Soil System: Effects and Mechanisms. Sci. Total Environ..

[52] Wang L., Li Z., Wang Y., Brookes P.C., Wang F., Zhang Q., Xu J., Liu X. (2021). Performance and Mechanisms for Remediation of Cd(II) and As(III) Co-Contamination by Magnetic Biochar-Microbe Biochemical Composite: Competition and Synergy Effects. Sci. Total Environ..

[53] Ali I., Suhail M., López E.C., Khattab R.A., Albishri H.M. (2022). Advances in Graphene-Based Materials for the Treatment of Water. Arab. J. Geosci..

[54] Tabish T.A., Memon F.A., Gomez D.E., Horsell D.W., Zhang S. (2018). A Facile Synthesis of Porous Graphene for Efficient Water and Wastewater Treatment. Sci. Rep..

[55] Akhtar M.S., Jutt D.S.R., Aslam S., Nawaz R., Irshad M.A., Khan M., Khairy M., Irfan A., Al-Hussain S.A., Zaki M.E.A. (2024). Green Synthesis of Graphene Oxide and Magnetite Nanoparticles and Their Arsenic Removal Efficiency from Arsenic Contaminated Soil. Sci. Rep..

[56] Baragaño D., Forján R., Welte L., Gallego J.L.R. (2020). Nanoremediation of As and Metals Polluted Soils by Means of Graphene Oxide Nanoparticles. Sci. Rep..

[57] Wei Y., Ma J., Liu K., Zhou T., Wang J. (2026). Structure-Performance Relationship of Graphene Aerogels in Heavy Metal Remediation: Advances in Preparation, Mechanisms, and Functionalization. Environ. Res..

[58] Gao Y., Li J., Li C., Chen H., Fang Z., Adusei-Fosu K., Wang Y., Trakal L., Wang H. (2024). A Novel Magnetic Graphene-Loaded Biochar Gel for the Remediation of Arsenic- and Antimony-Contaminated Mining Soil. Sci. Total Environ..

[59] Peña-Álvarez V., Baragaño D., Prosenkov A., Gallego J.R., Peláez A.I. (2024). Assessment of Co-Contaminated Soil Amended by Graphene Oxide: Effects on Pollutants, Microbial Communities and Soil Health. Ecotoxicol. Environ. Saf..

[60] Song Y., Lin W., Gustave W., Zhang Y., Feng D., Zhang X., He F. (2026). The Promises and Risks of Carbon-Based Nanomaterials: A Critical Review on Their Roles in Soil Health and Ecosystem Safety. Environ. Geochem. Health.

[61] Petrović J., Simić M., Ercegović M., Koprivica M., Dimitrijević J., Anđić D. (2025). Evaluating the Role of Hydrochars as Sustainable Adsorbents for Pollutant Removal. Metall. Mater. Data.

[62] Petrović J., Ercegović M., Simić M., Koprivica M., Dimitrijević J., Jovanović A., Janković Pantić J. (2024). Hydrothermal Carbonization of Waste Biomass: A Review of Hydrochar Preparation and Environmental Application. Processes.

[63] Lahori A.H., Ahmed S.R., Mierzwa-Hersztek M., Afzal M., Afzal A., Bano S., Muhammad M.T., Aqsa A., Vambol V., Vambol S. (2024). Comparative Role of Charcoal, Biochar, Hydrochar and Modified Biochar on Bioavailability of Heavy Metal(Loid)s and Machine Learning Regression Analysis in Alkaline Polluted Soil. Sci. Total Environ..

[64] Wang J., Geng Y., Hou H., Li X. (2025). Iron–Manganese-Modified Hydrochar for Synergistic Stabilization of Antimony and Arsenic in Smelter-Impacted Soils. Toxics.

[65] Lahori A.H., Mierzwa-Hersztek M., Lam N.T., Vambol S., Hussain M.I., Akbar S.A. (2026). Rock–Phosphate–Enriched Hydrochars Derived from Organic Wastes: A Sustainable Amendment for Multi-Metal Contaminated Soil. Waste Manag..

[66] Liu Z., Wang Z., Chen H., Cai T., Liu Z. (2021). Hydrochar and Pyrochar for Sorption of Pollutants in Wastewater and Exhaust Gas: A Critical Review. Environ. Pollut..

[67] Nguyen T.-H.A., Nakano A., Xuan T.D., Hoang L.K., Fumito M., Van Tam N., Hasan M.S., Johannes L.P., Van Thinh N. (2026). Dual-Route Synthesis of Bio-Composites for Rapid, High-Efficiency Arsenic and Lead Removal from Water: Comparing Pyrolysis and Hydrothermal Approaches. Bioresour. Technol. Rep..

[68] Na Y., Lee J., Lee S.H., Kumar P., Kim J.H., Patel R. (2020). Removal of Heavy Metals by Polysaccharide: A Review. Polym.-Plast. Technol. Mater..

[69] Ayub A., Raza Z.A. (2021). Arsenic Removal Approaches: A Focus on Chitosan Biosorption to Conserve the Water Sources. Int. J. Biol. Macromol..

[70] Wang J., Zhuang S. (2022). Chitosan-Based Materials: Preparation, Modification and Application. J. Clean. Prod..

[71] Padilla-Rodríguez A., Codling E.E. (2016). Potential of Chitosan (Chemically Modified Chitin) for Extraction of Lead-Arsenate Contaminated Soils. Commun. Soil Sci. Plant Anal..

[72] Xu Y., Huang X., Liu C., Kong D., Qian G. (2024). Chitosan-Modified Iron Fillings Materials for Remediation of Arsenic-Contaminated Soil. Chem. Eng. J..

[73] Hou X., Ma Z., Ke M., Zhou C., Zhang Y. (2025). Restoring Iron Mine Tailings-Contaminated Soils: A Biochar-Chitosan Composite Remediation Strategy. Alex. Eng. J..

[74] Yang J., Chen Z., Sun M., Xiao Y. (2026). Chitosan-Based Silicon Nanoparticles Inhibit Arsenic Uptake in Rice via Mediated Co-Deposition and Rhizosphere Microbiome Modulation. Plant Soil.

[75] Saiyad M., Shah N., Joshipura M., Dwivedi A., Pillai S. (2026). Modified Biopolymers in Wastewater Treatment: A Review. Mater. Today Proc..

[76] Zeng H., Ma G., Zheng X., Lin D., Zhang J., Li D. (2025). Preparation, Application and Regeneration of Chitosan Composite Adsorbents for Arsenic Removal: A Review from a Sustainable Perspective. Int. J. Biol. Macromol..

[77] Patel P.K., Pandey L.M., Uppaluri R.V.S. (2025). Conceptual Techno-Economic Analysis of Chitosan Derivative Resin and Commercial Sorbents for Multi-Heavy Metal Removal. Sep. Purif. Technol..

[78] Palanisamy K., Palanisamy G., Im Y.M., Thangarasu S., Phutela U.G., Oh T.H. (2025). Recent Progress in Cellulose-Based Aerogels for Sustainable Oil–Water Separation Technologies. Polymers.

[79] El Mahdaoui A., Radi S., Elidrissi A., Faustino M.A.F., Neves M.G.P.M.S., Moura N.M.M. (2024). Progress in the Modification of Cellulose-Based Adsorbents for the Removal of Toxic Heavy Metal Ions. J. Environ. Chem. Eng..

[80] Ibrahim H., Sazali N., Salleh W.N.W., Ismail A.F. (2021). Nanocellulose-Based Materials and Recent Application for Heavy Metal Removal. Water Air Soil Pollut..

[81] Yi X., Zhao H., Wei Y., Li Y., Wang T., Li Z., Kuang C., Yin K., Liu C. (2024). Sustainable Chloramine-Functionalized Iron Hydroxide Nanofiber Membrane for Arsenic(III) Removal via Oxidation-Adsorption Mechanism. Chemosphere.

[82] Shil R.K., Rahman I.M.M., Sakai Y., Marumoto M., Rocky M.M.H., Endo M., Wong K.H., Mashio A.S., Hasegawa H. (2025). Iron- and Zirconium-Modified Nanocellulose Adsorbent: Broad-Range Selectivity Test for Potentially Toxic Elements and Effective Arsenite Removal. Water Air Soil Pollut..

[83] Li Y., Wang J., Liu C., Wang L., Zhang P., Zhao Q., Xiong Z., Zhang G., Zhang W. (2024). Remediation of Arsenic-Contaminated Soil Using Nanoscale Schwertmannite Synthesized by Persulfate Oxidation with Carboxymethyl Cellulose Stabilization. Environ. Res..

[84] Su W., Xiao L. (2022). Manganese-Doped Ferrihydrite/Cellulose/Polyvinyl Alcohol Composite Membrane: Easily Recyclable Adsorbent for Simultaneous Removal of Arsenic and Cadmium from Soil. Sci. Total Environ..

[85] Marinho E. (2025). Cellulose: A Comprehensive Review of Its Properties and Applications. Sustain. Chem. Environ..

[86] Xiao D., Ding W., Zhang J., Ge Y., Wu Z., Li Z. (2019). Fabrication of a Versatile Lignin-Based Nano-Trap for Heavy Metal Ion Capture and Bacterial Inhibition. Chem. Eng. J..

[87] Wang T., Jiang M., Yu X., Niu N., Chen L. (2022). Application of Lignin Adsorbent in Wastewater Treatment: A Review. Sep. Purif. Technol..

[88] Zhang W., Duo H., Li S., An Y., Chen Z., Liu Z., Ren Y., Wang S., Zhang X., Wang X. (2020). An Overview of the Recent Advances in Functionalization Biomass Adsorbents for Toxic Metals Removal. Colloid Interface Sci. Commun..

[89] Shi X., Hong J., Li J., Kong S., Song G., Naik N., Guo Z. (2021). Excellent Selectivity and High Capacity of As (V) Removal by a Novel Lignin-Based Adsorbent Doped with N Element and Modified with Ca^2+^. Int. J. Biol. Macromol..

[90] Wu Q., Gao L., Huang M., Mersal G.A.M., Ibrahim M.M., El-Bahy Z.M., Shi X., Jiang Q. (2022). Aminated Lignin by Ultrasonic Method with Enhanced Arsenic (V) Adsorption from Polluted Water. Adv. Compos. Hybrid. Mater..

[91] Seo M., Cho W., Lee J.H., Park H., Lee J.Y. (2025). Evaluation of the Applicability of Iron Oxide-Modified Lignin as a Stabilizer for Arsenic-Contaminated Soil. J. Korea Soc. Waste Manag..

[92] Zhang Z., Chen Y., Wang D., Yu D., Wu C. (2023). Lignin-Based Adsorbents for Heavy Metals. Ind. Crops Prod..

[93] Tang H., Wang J., Zhang S., Pang H., Wang X., Chen Z., Li M., Song G., Qiu M., Yu S. (2021). Recent Advances in Nanoscale Zero-Valent Iron-Based Materials: Characteristics, Environmental Remediation and Challenges. J. Clean. Prod..

[94] Vítková M., Puschenreiter M., Komárek M. (2018). Effect of Nano Zero-Valent Iron Application on As, Cd, Pb, and Zn Availability in the Rhizosphere of Metal(Loid) Contaminated Soils. Chemosphere.

[95] Xie J., Wei H., Sun M., Huang L., Zhong J., Wu Y., Zou Q., Chen Z. (2024). The Performance and Mechanism of Sulfidated Nano-Zero-Valent Iron for the Simultaneous Stabilization of Arsenic and Cadmium. Sci. Total Environ..

[96] Jiang D., Zeng G., Huang D., Chen M., Zhang C., Huang C., Wan J. (2018). Remediation of Contaminated Soils by Enhanced Nanoscale Zero Valent Iron. Environ. Res..

[97] Alidokht L., Anastopoulos I., Ntarlagiannis D., Soupios P., Tawabini B., Kalderis D., Khataee A. (2021). Recent Advances in the Application of Nanomaterials for the Remediation of Arsenic-Contaminated Water and Soil. J. Environ. Chem. Eng..

[98] Chen A., Wang H., Zhan X., Gong K., Xie W., Liang W., Zhang W., Peng C. (2024). Applications and Synergistic Degradation Mechanisms of nZVI-Modified Biochar for the Remediation of Organic Polluted Soil and Water: A Review. Sci. Total Environ..

[99] Li Z., Wang L., Wu J., Xu Y., Wang F., Tang X., Xu J., Ok Y.S., Meng J., Liu X. (2020). Zeolite-Supported Nanoscale Zero-Valent Iron for Immobilization of Cadmium, Lead, and Arsenic in Farmland Soils: Encapsulation Mechanisms and Indigenous Microbial Responses. Environ. Pollut..

[100] Ainiwaer M., Jia H., Zhang T., Huang J., Zhang N., Yin X., Peng L., Li H., Zeng X. (2024). Effective Co-Immobilization of Arsenic and Cadmium in Contaminated Soil by Sepiolite-Modified Nano-Zero-Valent Iron and Its Impact on the Soil Bacterial Community. Sci. Rep..

[101] Gil-Díaz M., Rodríguez-Valdés E., Alonso J., Baragaño D., Gallego J.R., Lobo M.C. (2019). Nanoremediation and Long-Term Monitoring of Brownfield Soil Highly Polluted with As and Hg. Sci. Total Environ..

[102] An H., Liu T., Xiao X., Liu M., Hu Y., Wei P., Yao W., Tang X., Lai Y., Luo X. (2025). Magnetic Biochar-Supported Nanoscale Zero-Valent Iron for Remediation of Arsenic and Cadmium-Contaminated Soils: The Role of Free Radicals. Environ. Res..

[103] Liu K., Li F., Zhu Z., Fang L. (2024). Nanoconfined Fe(II) Releaser for Long-Term Arsenic Immobilization and Its Sustainability Assessment. Water Res..

[104] Liu H., Xie X., Cao H., Wang Y. (2023). Insights into the Selectivity of Metallic Oxides for Arsenic and Phosphate from EXAFS and DFT Calculations. Chemosphere.

[105] Barreto M.S.C., Elzinga E.J., Sparks D.L. (2023). The Adsorption of Arsenate and P-Arsanilic Acid onto Ferrihydrite and Subsequent Desorption by Sulfate and Artificial Seawater: Future Implications of Sea Level Rise. Environ. Pollut..

[106] Meng D., Nabi F., Kama R., Li S., Wang W., Guo Y., Li Z., Li H. (2024). The Interaction between Ferrihydrite and Arsenic: A Review of Environmental Behavior, Mechanism and Applied in Remediation. J. Hazard. Mater. Adv..

[107] Fischel M.H.H., Clarke C.E., Sparks D.L. (2024). Arsenic Sorption and Oxidation by Natural Manganese-Oxide-Enriched Soils: Reaction Kinetics Respond to Varying Environmental Conditions. Geoderma.

[108] Liang M., Guo H., Xiu W. (2024). Synergetic Effects of Mn(II) Production and Site Availability on Arsenite Oxidation and Arsenate Adsorption on Birnessite in the Presence of Low Molecular Weight Organic Acids. J. Hazard. Mater..

[109] Xu X., Chen C., Wang P., Kretzschmar R., Zhao F.-J. (2017). Control of Arsenic Mobilization in Paddy Soils by Manganese and Iron Oxides. Environ. Pollut..

[110] Wang Y., Tsang Y.F., Wang H., Sun Y., Song Y., Pan X., Luo S. (2020). Effective Stabilization of Arsenic in Contaminated Soils with Biogenic Manganese Oxide (BMO) Materials. Environ. Pollut..

[111] Baragaño D., Alonso J., Gallego J.R., Lobo M.C., Gil-Díaz M. (2020). Zero Valent Iron and Goethite Nanoparticles as New Promising Remediation Techniques for As-Polluted Soils. Chemosphere.

[112] Zhang T., Chen X., Wang Y., Li L., Sun Y., Wang Y., Zeng X. (2022). The Stability of Poorly Crystalline Arsenical Ferrihydrite after Long-Term Soil Suspension Incubation. Chemosphere.

[113] Huang Y., Liu Q., Luo J., Huang F., Yan X., Huang X. (2024). Silicate Impedes Arsenic Release and Oxidation from Ferrihydrite. J. Hazard. Mater..

[114] Kong S., Cai D., Shao Y., Wei X., Yi Z., Root R.A., Chorover J. (2024). Identification of Key Factors and Mechanism Determining Arsenic Mobilization in Paddy Soil-Porewater-Rice System. J. Hazard. Mater..

[115] Dong Z.-K., Han B.-L., Chen C., Gao A.-X., Tang Z., Zhao F.-J. (2025). Microbial Drivers Controlling Arsenic Mobilization in Paddy Soils and Arsenic Accumulation in Rice Grains. Environ. Pollut..

[116] Zheng Q., Hou J., Hartley W., Ren L., Wang M., Tu S., Tan W. (2020). As(III) Adsorption on Fe-Mn Binary Oxides: Are Fe and Mn Oxides Synergistic or Antagonistic for Arsenic Removal?. Chem. Eng. J..

[117] McCann C.M., Peacock C.L., Hudson-Edwards K.A., Shrimpton T., Gray N.D., Johnson K.L. (2018). In Situ Arsenic Oxidation and Sorption by a Fe-Mn Binary Oxide Waste in Soil. J. Hazard. Mater..

[118] Si M., Cao W., Ou C., Tu G., Yang W., Li Q., Liao Q., Yang Z. (2023). Simultaneous Oxidization-Adsorption for Arsenic and Antimony by Biological Fe-Mn Binary Oxides (BFMO): The Efficiencies and Mechanism. Process Saf. Environ. Prot..

[119] Li M., Kang Y., Kuang S., Wu H., Zhuang L., Hu Z., Zhang J., Guo Z. (2024). Efficient Stabilization of Arsenic Migration and Conversion in Soil with Surfactant-Modified Iron-Manganese Oxide: Environmental Effects and Mechanistic Insights. Sci. Total Environ..

[120] Zhang Y., Fu P., Li S., Deng W., Guo L., Li S., Wang X. (2024). Remediation of Multiple Heavy Metals Contaminated Soils by Mn and Fe-Added Solid Wastes: Effect and Mechanisms. Chem. Eng. J..

[121] Zhang Y., Fu P., Ni W., Zhang S., Li S., Deng W., Hu W., Li J., Pei F., Du L. (2024). A Review of Solid Wastes-Based Stabilizers for Remediating Heavy Metals Co-Contaminated Soil: Applications and Challenges. Sci. Total Environ..

[122] Gao W., Zhou W., Lyu X., Liu X., Su H., Li C., Wang H. (2023). Comprehensive Utilization of Steel Slag: A Review. Powder Technol..

[123] Raza M.B., Datta S.P., Golui D., Barman M., Ray P., Upadhyay D., Mishra R., Roy A., Dash A.K. (2024). Enhancing Soil Arsenic Immobilization with Organic and Inorganic Amendments: Insights from Sorption–Desorption Study. Environ. Monit. Assess..

[124] Zhang Y., Hou Z., Fu P., Wang X., Xue T., Chen Y. (2023). Remediation of As-Contaminated Soil by Utilizing Steel Slag-Based Passivators with Different Fe/Ca Ratios: As Bioavailability and Passivation Characterization. J. Clean. Prod..

[125] Derakhshan Nejad Z., Kim J.W., Jung M.C. (2017). Reclamation of Arsenic Contaminated Soils around Mining Site Using Solidification/Stabilization Combined with Revegetation. Geosci. J..

[126] Zhang Y., Fu P., Li S., Deng W., Li S., Ni W., Zhang S. (2025). Dual Regulation of As Release and Soil Environment by Fe(II) Assisted Steel Slag and Coal Fly Ash: Effects and Potential Mechanisms. J. Hazard. Mater..

[127] Yu S., Garrabrants A.C., DeLapp R.C., Hubner T., Thorneloe S.A., Kosson D.S. (2024). From Leaching Data to Release Estimates: Screening and Scenario Assessments of Electric Arc Furnace (EAF) Slag under Unencapsulated Use. J. Hazard. Mater..

[128] He Y., Pan J., Huang D., Sanford R.A., Peng S., Wei N., Sun W., Shi L., Jiang Z., Jiang Y. (2023). Distinct Microbial Structure and Metabolic Potential Shaped by Significant Environmental Gradient Impacted by Ferrous Slag Weathering. Environ. Int..

[129] Zhang Y., Fu P., Li S., Deng W., Ni W., Zhang S., Guo L., Li S., Wang X. (2024). Remediation of As, Sb, and Pb Co-Contaminated Mining Soils by Using Fe/C Based Solid Wastes: Synergistic Effects and Field Applications. Chem. Eng. J..

[130] Gao W., Ni W., Zhang Y., Li Y., Shi T., Li Z. (2020). Investigation into the Semi-Dynamic Leaching Characteristics of Arsenic and Antimony from Solidified/Stabilized Tailings Using Metallurgical Slag-Based Binders. J. Hazard. Mater..

[131] Zhao F.-J., McGrath S.P., Meharg A.A. (2010). Arsenic as a Food Chain Contaminant: Mechanisms of Plant Uptake and Metabolism and Mitigation Strategies. Annu. Rev. Plant Biol..

[132] Teasley W.A., Limmer M.A., Seyfferth A.L. (2017). How Rice (*Oryza sativa* L.) Responds to Elevated As under Different Si-Rich Soil Amendments. Environ. Sci. Technol..

[133] Chi Y., Peng L., Tam N.F., Lin Q., Liang H., Li W.C., Ye Z. (2022). Effects of Fly Ash and Steel Slag on Cadmium and Arsenic Accumulation in Rice Grains and Soil Health: A Field Study over Four Crop Seasons in Guangdong, China. Geoderma.

[134] Zhou Y., Cui Y., Yang J., Chen L., Qi J., Zhang L., Zhang J., Huang Q., Zhou T., Zhao Y. (2024). Roles of Red Mud in Remediation of Contaminated Soil in Mining Areas: Mechanisms, Advances and Perspectives. J. Environ. Manag..

[135] Jiang Q., He Y., Wu Y., Dian B., Zhang J., Li T., Jiang M. (2022). Solidification/Stabilization of Soil Heavy Metals by Alkaline Industrial Wastes: A Critical Review. Environ. Pollut..

[136] Mayes W.M., Burke I.T., Gomes H.I., Anton Á.D., Molnár M., Feigl V., Ujaczki É. (2016). Advances in Understanding Environmental Risks of Red Mud after the Ajka Spill, Hungary. J. Sustain. Metall..

[137] Jalayer A., Ashrafi K., Ghazban F., Hosseinalizadeh M. (2026). Modeling Dispersion of Dust Caused by Tailings and Mining Activities at Jajarm Based on Gradation Analysis. Environ. Monit. Assess..

[138] Yang D., Chu Z., Feng X., Ge Q., Wang R., Zhang J., Li S., Zheng R., Wei W., Yi S. (2022). Dual Ions Neutralized and Stabilized Red Mud for Chromium(VI) Polluted Soil Remediation. ACS EST Eng..

[139] Lopes G., Guilherme L.R.G., Costa E.T.S., Curi N., Penha H.G.V. (2013). Increasing Arsenic Sorption on Red Mud by Phosphogypsum Addition. J. Hazard. Mater..

[140] De Souza Costa E.T., Lopes G., Carvalho G.S., Penha H.G.V., Curi N., Guilherme L.R.G. (2021). Phytoremediation of Arsenic-Contaminated Soils Amended with Red Mud Combined with Phosphogypsum. Water Air Soil Pollut..

[141] Zhang Y., Tan X., Duan G., Cui J., Ren M., Cao J., Xu C., Yang W., Lin A. (2022). Magnesium Slag for Remediation of Cadmium- and Arsenic-Contaminated Paddy Soil: A Field Study. Soil Use Manag..

[142] Ha Z., Ma M., Tan X., Lan Y., Lin Y., Zhang T.C., Du D. (2023). Remediation of Arsenic Contaminated Water and Soil Using Mechanically (Ball Milling) Activated and Pyrite-Amended Electrolytic Manganese Slag. Environ. Res..

[143] Yan Y., Sun S., Shubo A., Wei J., Xiao F., Tu G. (2026). Towards Sustainable Management of Electrolytic Manganese Residue: Pollution Control and Circular Resource Utilization. Miner. Eng..

[144] Liu J., Du Y., Liu Y., Wang J., Zhang W. (2026). Volume Stability of Magnesium Slag-Based Building Materials: A Critical Review of Mechanisms and Mitigation Strategies. Materials.

[145] Gao L., Liu Y., Xu K., Bai L., Guo N., Li S. (2024). A Short Review of the Sustainable Utilization of Coal Gangue in Environmental Applications. RSC Adv..

[146] Zheng Q., Zhou Y., Liu X., Liu M., Liao L., Lv G. (2024). Environmental Hazards and Comprehensive Utilization of Solid Waste Coal Gangue. Prog. Nat. Sci. Mater. Int..

[147] Chen M., Liu Y., Zhang D., Zhu J., Chen X., Yuan L. (2022). Remediation of Arsenic-Contaminated Paddy Soil by Iron Oxyhydroxide and Iron Oxyhydroxide Sulfate-Modified Coal Gangue under Flooded Condition. Sci. Total Environ..

[148] Chen M., Zhou Y., Sun Y., Chen X., Yuan L. (2023). Coal Gangue-Based Magnetic Porous Material for Simultaneous Remediation of Arsenic and Cadmium in Contaminated Soils: Performance and Mechanisms. Chemosphere.

[149] Xu S., Yang Y., Li W., Lu Y., Yang H., Huang Q., Die Q. (2026). Leaching Mechanisms of Potentially Toxic Elements from Coal Gangue: Thermodynamic, Kinetic, and Colloidal Effects. Environ. Pollut..

[150] Nash W., Fahmi R., Ramos V., Crane R. (2025). Assessment of Ecotoxic Metal Leaching Behaviour of Coal Gangue Using a Sequential Extraction Procedure. Mine Water Environ..

[151] Tao H., Qian X., Zhou Y., Cheng H. (2022). Research Progress of Clay Minerals in Carbon Dioxide Capture. Renew. Sustain. Energy Rev..

[152] Yang X., Zhou Y., Hu J., Zheng Q., Zhao Y., Lv G., Liao L. (2024). Clay Minerals and Clay-Based Materials for Heavy Metals Pollution Control. Sci. Total Environ..

[153] Xu R., Li Q., Yang Y., Jin S., Liao L., Wu Z., Yin Z., Xu B., Nan X., He Y. (2022). Removal of Heavy Metal(Loid)s from Aqueous Solution by Biogenic FeS–Kaolin Composite: Behaviors and Mechanisms. Chemosphere.

[154] Wang L., Cho D.-W., Tsang D.C.W., Cao X., Hou D., Shen Z., Alessi D.S., Ok Y.S., Poon C.S. (2019). Green Remediation of As and Pb Contaminated Soil Using Cement-Free Clay-Based Stabilization/Solidification. Environ. Int..

[155] Zhou X., Zhang Z., Yang H., Bao C., Wang J., Sun Y., Liu D., Shen P., Su C. (2021). Red Mud-Metakaolin Based Cementitious Material for Remediation of Arsenic Pollution: Stabilization Mechanism and Leaching Behavior of Arsenic in Lollingite. J. Environ. Manag..

[156] Zhao C., Yao J., Knudsen T.Š., Liu J., Zhu X., Ma B. (2023). Effect of Goethite-Loaded Montmorillonite on Immobilization of Metal(Loid)s and the Micro-Ecological Soil Response in Non-Ferrous Metal Smelting Areas. Sci. Total Environ..

[157] Arabmofrad S., Lazzara G., Miller R., Jafari S.M. (2024). Surface Modification of Bentonite and Montmorillonite as Novel Nano-Adsorbents for the Removal of Phenols, Heavy Metals and Drug residuesBentonite. Adv. Colloid Interface Sci..

[158] Dousova B., Lhotka M., Filip J., Kolousek D. (2018). Removal of Arsenate and Antimonate by Acid-Treated Fe-Rich Clays. J. Hazard. Mater..

[159] Mukhopadhyay R., Manjaiah K.M., Datta S.C., Yadav R.K., Sarkar B. (2017). Inorganically Modified Clay Minerals: Preparation, Characterization, and Arsenic Adsorption in Contaminated Water and Soil. Appl. Clay Sci..

[160] Guo Y.X., Liu J.H., Gates W.P., Zhou C.H. (2020). Organo-Modification of Montmorillonite. Clays Clay Miner..

[161] Li Q., Sun X., Zhang W., Sun Z., Na S., Chen Z., Wang L., Yuan C., Sun H. (2022). Effect of Fe(III)-Modified Montmorillonite on Arsenic Oxidation and Anthracene Transformation in Soil. Sci. Total Environ..

[162] Zhao C., Yao J., Knudsen T.Š., Liu J., Zhu X., Ma B., Li H., Cao Y., Liu B. (2023). Performance and Mechanisms for Cd(II) and As(III) Simultaneous Adsorption by Goethite-Loaded Montmorillonite in Aqueous Solution and Soil. J. Environ. Manag..

[163] Jiang M., Wang K., Li G., Zhao Q., Wang W., Jiang J., Wang Y., Yuan L. (2023). Stabilization of Arsenic, Antimony, and Lead in Contaminated Soil with Montmorillonite Modified by Ferrihydrite: Efficiency and Mechanism. Chem. Eng. J..

[164] Yu K., Xu J., Jiang X., Liu C., McCall W., Lu J. (2017). Stabilization of Heavy Metals in Soil Using Two Organo-Bentonites. Chemosphere.

[165] Hacıosmanoğlu G.G., Mejías C., Martín J., Santos J.L., Aparicio I., Alonso E. (2022). Antibiotic Adsorption by Natural and Modified Clay Minerals as Designer Adsorbents for Wastewater Treatment: A Comprehensive Review. J. Environ. Manag..

[166] Wang J., Sun Q., Gao Q., Zheng H., He J., Jiang Y., Liu Z., Zhang W. (2022). Effective Immobilization of Arsenic in Waters and Sediments Using Novel Zirconium-Loaded Lanthanum-Modified Bentonite Capping. J. Environ. Chem. Eng..

[167] Fakhreddine S., Fendorf S. (2021). The Effect of Porewater Ionic Composition on Arsenate Adsorption to Clay Minerals. Sci. Total Environ..

[168] Wang X., Pu S., Ding J., Chen J., Liao P., Zhong D., Tsang D.C.W., Crittenden J.C., Wang L. (2024). Enhanced Arsenate Immobilization by Kaolinite via Heterogeneous Pathways during Ferrous Iron Oxidation. Environ. Sci. Technol..

[169] Fu T., Shi J., Wang X., Tang L., Yang Z., Chen B., Zhang Y., Zhang B. (2026). From Natural Adsorption to Targeted Immobilization: Efficacy and Mechanisms of Clay Minerals in Remediating Soil Heavy Metals. J. Hazard. Mater. Adv..

[170] Tang C., Yao J., Lv Y., Liu J., Ma B., Knudsen T.Š., Liu B., Yu J., Liu X. (2026). Fe-Modified Clay Minerals Enhances Iron Oxide Transformation and Microbial Ecological Succession for Simultaneous Stabilization of As, Pb, and Cd in Smelting Soils. Environ. Res..

[171] Kang L., Xu B., Li P., Wang K., Chen J., Du H., Liu Q., Zhang L., Lian X. (2025). Controllable Preparation of Low-Cost Coal Gangue-Based SAPO-5 Molecular Sieve and Its Adsorption Performance for Heavy Metal Ions. Nanomaterials.

[172] Velarde L., Nabavi M.S., Escalera E., Antti M.-L., Akhtar F. (2023). Adsorption of Heavy Metals on Natural Zeolites: A Review. Chemosphere.

[173] Mendoza-Barrón J., Jacobo-Azuara A., Leyva-Ramos R., Berber-Mendoza M.S., Guerrero-Coronado R.M., Fuentes-Rubio L., Martínez-Rosales J.M. (2011). Adsorption of Arsenic (V) from a Water Solution onto a Surfactant-Modified Zeolite. Adsorption.

[174] Figueiredo H., Quintelas C. (2014). Tailored Zeolites for the Removal of Metal Oxyanions: Overcoming Intrinsic Limitations of Zeolites. J. Hazard. Mater..

[175] Wen J., Yan C., Xing L., Wang Q., Yuan L., Hu X. (2021). Simultaneous Immobilization of As and Cd in a Mining Site Soil Using HDTMA-Modified Zeolite. Environ. Sci. Pollut. Res. Int..

[176] Yang D., Ge Q., Feng X., Wang R., Li S., Wei W., Zheng R., Zhang J., Chen H. (2023). “Soil for Soil Remediation” Strategy Driven on Converting Natural Soils into Fe2O3-CAN-Type Zeolite Composites for Dual Ionic Heavy Metal-Contaminated Soil Remediation: Universality, Synergistic Effects, and Mechanism. ACS EST Eng..

[177] Zheng R., Feng X., Zou W., Wang R., Yang D., Wei W., Li S., Chen H. (2021). Converting Loess into Zeolite for Heavy Metal Polluted Soil Remediation Based on “Soil for Soil-Remediation” Strategy. J. Hazard. Mater..

